# Supplementary material for: Study environment and the incidence of mental health problems and activity-limiting musculoskeletal problems among university students: the SUN cohort study
Source: BMJ Open. 2023 Sep 14;13(9):e072178. doi: 10.1136/bmjopen-2023-072178 (PMC10503358; doi:10.1136/bmjopen-2023-072178)
Supplement: Supplementary data [file bmjopen-2023-072178supp001.pdf]

Supplemental material

Johansson, F., Billquist, J., Andreasson, H., Jensen, I., Onell, C., Berman, A. H., Skillgate, E. Study Environment and the Incidence of Mental Health Problems and Activity-Limiting Musculoskeletal Problems among University Students: The SUN Cohort Study

eTable 1. Number of events and participants at risk at each time-period.

eTable 2. COVID-19 adjusted hazard ratios of mental health problems and activity-limiting musculoskeletal problems in any body location comparing exposed to unexposed

eTable 3. Gender specific hazard ratios of mental health problems and activity-limiting musculoskeletal problems in any body location comparing exposed to unexposed

eTable 4. Association between baseline exposure and dropout at the 12-month follow-up

eTable 5. E-values for selection bias giving the minimum association between the outcome and missingness among the unexposed needed to move the HR estimates to the null

eTable 6. Dose response models for high study pace and low social cohesion with each response category as separate exposure level

eTable 1. Number of events and participants at risk at each time-period

	Mental health cohort			Musculoskeletal health cohort		
Time-period	*Events (n)*	*At risk (n)* [a]	*Hazard* [b]	*Events (n)*	*At risk (n)* [a]	*Hazard* [b]
0-3 months	502	2087	0.24	475	2361	0.20
3-6 months	180	1436	0.13	234	1711	0.13
6-9 months	133	1163	0.11	142	1357	0.10
9-12 months	94	997	0.09	121	1184	0.10

[a] Number are not equal to the analytic sample since not all participants were observed at all time-points.

[b] The hazard equals the probability of the event conditional on not having had the event earlier (Events / At risk).

eTable 2. COVID-19 adjusted [a] hazard ratios of mental health problems and activity-limiting musculoskeletal problems in any body location comparing exposed to unexposed

	**Mental health problems**	**Activity-limiting musculoskeletal problems**
	Adjusted HR [b] *(95% CI)*	*Adjusted HR [b]* *(95% CI)*
Discrimination	1.76 (1.41 – 2.21)	1.38 (1.12 – 1.72)
High study pace	1.70 (1.48 – 1.95)	1.25 (1.09 – 1.43)
Low social cohesion	1.51 (1.29 – 1.76)	1.08 (0.93 – 1.26)
Poor physical environment	1.20 (0.99 – 1.46)	1.20 (1.01 – 1.43)

[a] these analyses include a dummy coded covariate indicating whether the baseline survey, where the exposures were measured, were collected before or after March 13, 2020, that is considered the start of the COVID-19 pandemic in Sweden.

[b] Adjusted for gender, age, living situation, education type, year of study, place of birth and highest level of parental education and covid-19 pandemic at baseline.

eTable 3. Gender specific hazard ratios of mental health problems and activity-limiting musculoskeletal problems in any body location comparing exposed to unexposed

	**Mental health problems** *Adjusted HR* [a] *(95% CI)*		**Activity-limiting musculoskeletal problems** *Adjusted HR* [a] *(95% CI)*	
	Women (n = 1263 [b])	Men (n = 927 [c])	Women (n = 1491 [d])	Men (n = 1007 [e])
Discrimination	1.79 (1.39 – 2.31)	1.85 (1.16 – 2.95)	1.31 (1.01 - 1.69)	1.58 (1.08 – 2.33)
High study pace	1.50 (1.27 – 1.78)	2.11 (1.67 – 2.66)	1.29 (1.09 - 1.53)	1.15 (0.92 – 1.43)
Low social cohesion	1.40 (1.15 – 1.71)	1.81 (1.41 – 2.32)	1.14 (0.94 – 1.38)	1.01 (0.79 – 1.29)
Poor physical environment	1.12 (0.89 – 1.42)	1.31 (0.94 – 1.83)	1.18 (0.95 – 1.46)	1.28 (0.95 – 1.71)

[a] Adjusted for age, living situation, education type, year of study, place of birth and highest level of parental education.

[b] 1427 women were included in the cohort at baseline, but 164 did not provide any follow-up assessments, giving an analytic sample size of 1263.

[c] 1065 men were included in the cohort at baseline, but 138 did not provide any follow-up assessments, giving an analytic sample size of 927.

[d] 1698 women were included in the cohort at baseline, but 206 did not provide any follow-up assessments, giving an analytic sample size of 1491

[e] 1057 men were included in the cohort at baseline, but 150 did not provide any follow-up assessments, giving an analytic sample size of 1007.

eTable 4. Association between baseline exposure and dropout at the 12-month follow-up

	**Mental health cohort**	**Musculoskeletal health cohort**
	RR (95% CI)	*RR (95% CI)*
Discrimination	1.15 (0.95 - 1.39)	1.27 (1.10 – 1.48)
High study pace	0.98 (0.88 – 1.09)	1.03 (0.94 – 1.14)
Low social cohesion	1.05 (0.92 – 1.20)	1.07 (0.96 – 1.19)
Poor physical environment	1.10 (0.95 – 1.28)	1.18 (1.04 – 1.33)

RR: Risk ratio

eTable 5. E-values for selection bias giving the minimum association between the outcome and missingness among the unexposed needed to move the HR estimates [a] to the null [b]

	**Mental health cohort**	**Musculoskeletal health cohort**
	RR (95% CI)	*RR (95% CI)*
Discrimination	1.47	1.26
High study pace	1.44	1.16
Low social cohesion	1.33	1.05
Poor physical environment	1.13	1.13

[a] This refers to the adjusted estimates presented in Table 5
[b] Assuming that missingness is higher for persons with the outcome among both exposed and unexposed
RR: Risk ratio

eTable 6. Dose response models for high study pace and low social cohesion with each response category as a separate exposure level

	Mental health cohort		Musculoskeletal health cohort	
	No. exp [a]	*Adjusted HR (95% CI)* [b]	*No. exp* [a]	*Adjusted HR (95% CI)* [b]
**High study pace**				
I feel that I have energy and time to cope with the study pace	1079	Ref.	1044	Ref.
The study pace is high. I do my best, but I am afraid that it is not enough	967	1.64 (1.43 - 1.88)	1201	1.20 (1.05 - 1.37)
The study pace breaks me. We get more assignments than I have time for, and I feel that I cannot make it	41	3.28 (2.25 - 4.77)	116	1.50 (1.13 - 1.98)
**Low social cohesion**				
The cohesion is pretty good. We help each other with the studies and there is always someone in the class to talk to	1662	Ref.	1779	Ref.
I do not think that our cohesion is good. We work independently, and it feels like it is not worth the inconvenience to try to change it	366	1.49 (1.27 - 1.70)	506	1.07 (0.92 - 1.25)
We do not have any cohesion, and no one takes any responsibility over our groupwork or the fellowship	26	2.12 (1.30 - 3.45)	45	1.14 (0.72 - 1.80)

[a] Refers to the number of exposed participants in the analytic sample at baseline.
[b] Adjusted for age, living situation, education type, year of study, place of birth and highest level of parental education.
