# Supplementary material for: Study environment and the incidence of mental health problems and activity-limiting musculoskeletal problems among university students: the SUN cohort study
Source: BMJ Open. 2023 Sep 14;13(9):e072178. doi: 10.1136/bmjopen-2023-072178 (PMC10503358; doi:10.1136/bmjopen-2023-072178)
Supplement: Author's manuscript [file bmjopen-2023-072178.draft_revisions.pdf]

BMJ Open

BMJ Open is committed to open peer review. As part of this commitment we make the peer review history of every article we publish publicly available.

When an article is published we post the peer reviewers' comments and the authors' responses online. We also post the versions of the paper that were used during peer review. These are the versions that the peer review comments apply to.

The versions of the paper that follow are the versions that were submitted during the peer review process. They are not the versions of record or the final published versions. They should not be cited or distributed as the published version of this manuscript.

BMJ Open is an open access journal and the full, final, typeset and author-corrected version of record of the manuscript is available on our site with no access controls, subscription charges or pay-per-view fees (http://bmjopen.bmj.com).

If you have any questions on BMJ Open's open peer review process please email info.bmjopen@bmj.com

BMJ Open

Study Environment and the Incidence of Mental Health Problems and Disabling Musculoskeletal Pain among University Students: The SUN Cohort Study

Journal:	*BMJ Open*
Manuscript ID	bmjopen-2023-072178
Article Type:	Original research
Date Submitted by the Author:	25-Jan-2023

Complete List of Authors:	Johansson, Fred; Sophiahemmet University, Department of Health Promotion Science<br>Billquist, Jessica; Uppsala Universitet, Department of Psychology<br>Andreasson, Hanna; Uppsala Universitet, Department of Psychology<br>Jensen, Irene; Karolinska Institutet, Institute of Environmental Medicine<br>Onell, Clara; Sophiahemmet University, Department of Health Promotion Science<br>Berman, Anne H.; Uppsala Universitet, Department of Psychology; Karolinska Institutet, Department of Clinical Neuroscience<br>Skillgate, Eva; Sophiahemmet University, Department of Health Promotion Science; Karolinska Institutet, Institute of Environmental Medicine

Keywords:	MENTAL HEALTH, Depression & mood disorders < PSYCHIATRY, Musculoskeletal disorders < ORTHOPAEDIC & TRAUMA SURGERY, EPIDEMIOLOGY

SCHOLARONE™
Manuscripts

I, the Submitting Author has the right to grant and does grant on behalf of all authors of the Work (as defined in the below author licence), an exclusive licence and/or a non-exclusive licence for contributions from authors who are: i) UK Crown employees; ii) where BMJ has agreed a CC-BY licence shall apply, and/or iii) in accordance with the terms applicable for US Federal Government officers or employees acting as part of their official duties; on a worldwide, perpetual, irrevocable, royalty-free basis to BMJ Publishing Group Ltd ("BMJ") its licensees and where the relevant Journal is co-owned by BMJ to the co-owners of the Journal, to publish the Work in this journal and any other BMJ products and to exploit all rights, as set out in our licence.

The Submitting Author accepts and understands that any supply made under these terms is made by BMJ to the Submitting Author unless you are acting as an employee on behalf of your employer or a postgraduate student of an affiliated institution which is paying any applicable article publishing charge ("APC") for Open Access articles. Where the Submitting Author wishes to make the Work available on an Open Access basis (and intends to pay the relevant APC), the terms of reuse of such Open Access shall be governed by a Creative Commons licence – details of these licences and which Creative Commons licence will apply to this Work are set out in our licence referred to above.

Other than as permitted in any relevant BMJ Author's Self Archiving Policies, I confirm this Work has not been accepted for publication elsewhere, is not being considered for publication elsewhere and does not duplicate material already published. I confirm all authors consent to publication of this Work and authorise the granting of this licence.

For peer review only

Study Environment and the Incidence of Mental Health Problems and Disabling Musculoskeletal Pain among University Students: The SUN Cohort Study

Authors

Fred Johansson [1*]

Jessica Billquist[2]

Hanna Andreasson[2]

Irene Jensen[3]

Clara Onell [1]

Anne H Berman[2,4]

Eva Skillgate [1,3,5]

[1]Department of Health Promotion Science, Sophiahemmet University, Stockholm, Sweden

[2]Department of Psychology, Uppsala University, Uppsala, Sweden

[3]Unit of Intervention and Implementation Research for Worker Health, Institute of Environmental Medicine, Karolinska Institutet, Stockholm, Sweden

[4]Department of Clinical Neuroscience, Karolinska Institutet, Stockholm, Sweden

[5]Naprapathögskolan – Scandinavian College of Naprapathic Manual Medicine, Stockholm, Sweden

* Corresponding author: Fred Johansson, Sophiahemmet Högskola, Box 5605, 114 86 Stockholm, Sweden. Email: fred.johansson@shh.se. Phone: +46739516158

Word count: 3123

Keywords: Students; Mental Health; Musculoskeletal Pain; Perceived Discrimination; Social Cohesion; Stress, Psychological

For peer review only

Abstract

Objective. To determine the association between different aspects of study environment and the incidence of mental health problems and disabling musculoskeletal pain, respectively. **Design, setting and participants.** In this cohort study**,** we recruited 4262 Swedish university students of whom 2503 (59%) were without moderate or worse mental health problems and 2889 (68%) disabling pain at baseline. The participants were followed at five time-points over on year using web surveys. **Exposures.** Self-rated discrimination, high study pace, low social cohesion and poor physical environment. **Outcomes:** Self-rated mental health problems, defined as scoring above cut-off on any of the subscales of the Depression, anxiety, and stress scale. Self-rated disabling musculoskeletal pain in any bodily location, assessed by the Nordic Musculoskeletal Questionnaire. **Statistical analysis.** Discrete survival-time analysis was used to estimate the hazard rate ratio (HR) of each exposure-outcome combination while adjusting for gender, age, living situation, education type, year of studies, place of birth and parental education as potential confounders. **Results.** For discrimination, the adjusted HRs were 1.75 (95% CI: 1.40 – 2.19) for mental health problems and 1.34 (95% CI: 1.08 – 1.67) for disabling pain. For high study pace, the adjusted HRs were 1.70 (95% CI: 1.48 – 1.94) for mental health problems and 1.24 (95% CI: 1.09 – 1.42) for disabling pain. For low social cohesion, the adjusted HRs were 1.51 (95% CI: 1.29 – 1.77) for mental health problems and 1.09 (95% CI: 0.93 – 1.26) for disabling pain. For perceived poor physical study environment, the adjusted HRs were 1.20 (95% CI: 0.99 – 1.45) for mental health problems and 1.23 (95% CI: 1.04 – 1.47) for disabling pain. **Conclusions.** Several aspects of the study environment were associated with the incidence of mental health problems and disabling pain in this sample of Swedish university students.

For peer review only

Strengths and limitations of this study

- We recruited a large sample of students, which enables us to estimate associations between the exposures and the outcomes with good precision.
- We followed the participants over time, and were able to adjust for multiple potential confounders, which strengthens the evidence level for causal associations.
- There may still be residual and unmeasured confounding that could affect the associations.
- Exposures were measured with single items questions, which could increase the risk of misclassification.
- The sample is not representative of Swedish university students overall, which may affect generalizability. We had some loss to follow-up, which could lead to selection bias that could affect the estimated associations.

For peer review only

Introduction

Mental health problems and musculoskeletal pain are leading causes of years lost due to disability in young people globally (1), and common among university students (2-5). Previous studies show that 35.7% of students from high-income countries meet criteria for at least one common mental disorder (5). Mental health problems are associated with increased risk of student attrition (5), academic impairment (6), reduced occupational preparedness and future work performance (7). Musculoskeletal pain is also common among university students (8-10) and associated with poorer academic functioning and lower quality of life (11).

Mental health problems and pain commonly co-occur (12). This comorbidity has been suggested to be characterized by bidirectional causation, with pain influencing mental health and vice versa (13, 14). Given the high comorbidity and bidirectional influences, it is likely that these conditions share many risk factors. Some risk factors are likely to affect students and non-students alike. Others, like the exposure to university study environments, are specific to students, and pose an important research target for understanding and preventing student health problems. Study environment is a multifaceted construct with several physical and psychosocial aspects. This study focuses on four specific aspects: discrimination, study pace, social cohesion, and physical study environment.

Seven percent of Swedish students report experiencing discrimination during their studies (15). Perceived discrimination is associated with mental and physical ill health (16) and is negatively associated with subsequent psychological well-being even after controlling for baseline levels of psychological well-being (17). Brown et al (18) showed that the association between discrimination and chronic pain is mediated through psychological distress in a general population sample. However, little is known as to how discrimination occurring specifically at the university affects students' health.

Perceived stress and academic demands have been associated with higher levels of depressive symptoms and psychological distress among students in longitudinal studies (19, 20). Perceived

For peer review only

work/study demands with neck and upper back pain among young adults (21). However, few studies have focused specifically on high study pace and its associations to mental health and disabling pain.

Social isolation and loneliness are well-established risk factors for mental health problems in general (22), but less is known about the impact of low social cohesion within the study environment. For adolescents, a lack of social cohesion at school and lack of classmate support are cross-sectionally associated with lower psychological well-being and higher symptom levels of depression and anxiety (23). Low workplace social support has also been identified as a risk factor for back pain among working adults (24), but to our knowledge there is no studies on the association between social cohesion and pain among university students.

Physical aspects of the study environment such as repetitive movement, prolonged sitting and sitting position are associated with musculoskeletal pain among university students (25) and school children (26). However, there is a lack of research into how students' perceptions of physical study environment are associated with subsequent mental health problems and musculoskeletal pain.

In this study, we aimed to determine the associations between four aspects of study environment: discrimination; high study pace; low social cohesion; and perceived poor physical environment, and the incidence of mental health problems and disabling musculoskeletal pain among Swedish university students.

For peer review only

Method

Design and participants

This study is based on the Sustainable UNiversity life (SUN) cohort (27), a longitudinal study aiming to identify risk and prognostic factors for mental health problems and musculoskeletal pain in university students. Participants were recruited between August 2019 and December 2021, from eight universities in the greater Stockholm area and Örebro. The targeted universities constitute a convenience sample, selected to cover a variety of educational programs mainly within medicine,

business, technology, and social sciences. Full-time undergraduate or graduate students (up to and including masters' level) at selected educational programs with at least one full year of academic studies left before graduation were eligible for inclusion.

Eligible students were informed about the study during an in-class presentation and/or by email with a link to the baseline survey. Students agreeing to participate were followed every three months for one year using web surveys. For the aim of studying incidence of mental health problems and disabling musculoskeletal pain in this study, two cohorts were established: 1) the mental health cohort (MHC) including participants without moderate or more severe mental health problems at baseline; and 2) the musculoskeletal health cohort (MSKHC) including participants without disabling musculoskeletal pain at baseline (Figure 1).

The study was approved by the Swedish Ethical Review Authority (2019-03276), and all participants provided informed consent electronically before entering the study. More information about the study methodology and data collection is available in the study protocol (27).

Patient and public involvement

We involved students by conducting focus group interviews on health challenges of university students during the planning phase of the study. We have also had an ongoing dialogue with student health services, educational offices and student unions during the planning and conduct of the study, as well as for dissemination of the results. Students and university officials have been involved in the selection of exposures and outcomes, in recruitment and data collection phase, and have been offered to take part of the results of the study (for instance by participating in seminars organized by the research group).

Measures

Outcomes

Mental health problems were measured with the short-form Depression Anxiety Stress Scale (DASS-21) (28), consisting of 21 items including subscales on depression, anxiety, and stress. Each subscale

For peer review only

comprises seven items rated on a scale from 0 (Did not apply to me at all) to 3 (Applied to me very much, or most of the time), with a recall time of one week. The items were summed to give subscale-scores ranging from 0 to 21. Participants scoring above the cut-off for moderate symptoms levels on any of the scales (≥ 7 on the depression subscale, ≥ 6 on the anxiety subscale, ≥ 10 on the stress subscale) were considered to have mental health problems. DASS-21 has shown good psychometric properties among Swedish university students (29).

Disabling musculoskeletal pain in was measured with the Nordic Musculoskeletal Questionnaire (NMQ) (30). Participants were asked if they *"at any time during the last three months have been prevented from doing your normal activities (e.g. work, studies, household activities, hobbies) due to trouble with:"* ankles/feet, knee, hip, lower back, upper back, shoulder, elbow, hand, and/or neck. The question was responded to dichotomously (Yes/No) for each body location. Participants responding Yes for any of the body locations were considered to have disabling pain.

Exposures

All exposures were measured at baseline.

Discrimination was measured by the question: *"Have you ever during the past year felt discriminated by other students, teachers or administrative personnel due to:"* over nine categories: gender, gender identity, gender expression, ethnicity, religion or beliefs, sexual orientation, disabilities, age, other [15]. Participants answering "Yes" on at least one of the categories were asked to rate how severely the discrimination had affected them on a scale from 0 (Not at all) to 10 (Worst imaginable way). Participants with ratings >3 were considered exposed to discrimination.

Study pace was measured by the questions: "*Do you feel that you are coping with the study pace?"* with three response categories: (1) *"I feel that I have energy and time to cope with the study pace",* (2) "*The study pace is high. I do my best, but I am afraid that it is not enough"* and (3) "*The study pace breaks me. We get more assignments than I have time for, and I feel that I cannot make it"*. Participant's choosing categories 2 or 3 were considered exposed to high study pace.

Social cohesion was measured by the question: *"How good is the cohesion with your course mates?"*, with four response alternatives: (1) *"The cohesion is pretty good. We help each other with the studies and there is always someone in the class to talk to*", (2) *"I do not think that our cohesion is good. We work independently, and it feels like it is not worth the inconvenience to try to change it*", (3) *"We do not have any cohesion, and no one takes any responsibility over our groupwork or the fellowship*" and (4) *"My education is not organized around study groups or classes"*. Participants choosing categories 2 or 3 were considered exposed to low social cohesion. Participants choosing the fourth category were excluded from the risk analyses.

Perceived physical environment was measured by asking: "*How do you in general experience the physical study environment at your university? (For example, public areas, lunchroom, lecture halls, toilets, outside environment around campus.)"* and rated on a scale from 0 (Very bad) to 10 (Very good). Participants with ratings of 0-5 were considered exposed to a poor physical study environment.

Covariates

Potential confounders were selected based on previous research (e.g. (4, 31-33)) and directed acyclic graphs (34). We included the covariates gender (female, male, other), age (continuous), living situation (alone, with others), education type (medical/health, technical, social sciences/humanities, economic, other), year of studies (first, second, above), place of birth (Sweden, Scandinavia, Europe, outside Europe) and highest level of parental education (university, below university) as a proxy for socioeconomic status.

Statistical analysis

We used discrete-time survival analysis to estimate associations between baseline exposures and the hazard of each of the outcomes. A participant was considered at risk for the outcome until the outcome was first registered or until censoring due to loss to follow-up or end of follow-up, whichever came first.

We used generalized linear models with binomial error distributions and complementary log-log links to estimate the hazard ratio (HR) of the outcomes comparing the exposed to the unexposed. Separate models were built for each exposure – outcome combination. This modeling approach is equivalent to Cox proportional hazard models when assuming that the underlying time variable is continuous (35). Time was measured as four time-periods (0 to 3 months, 3 to 6 months, 6 to 9 months, and 9 to 12 months). Plotting the log hazard over the four time-periods showed a fairly linear relationship for both outcomes, and therefore the time-period variable was treated as continuous. The proportional hazard assumption was evaluated by including interaction terms between time-period and the exposures and performing likelihood ratio tests comparing models with and without interaction terms. These indicated better fits for models assuming proportional hazards for all exposure – outcome combinations, except for the model estimating the association between discrimination and mental health problems.

Sensitivity analyses

Subclinical symptom levels of mental health problems at baseline were assumed to be on the causal pathway and thus not included as covariates in the main analyses. To assess the sensitivity of our analyses to this assumption, we performed sensitivity analyses where the continuous scores of the three DASS-21 subscales were included as covariates.

All analyses were performed using R version 4.1.2.

Results

We included 4262 participants, of whom 2503 (59%) were without moderate or worse mental health problems at baseline and were included in the MHC and 2889 (68%) were without disabling pain in any body location at baseline and included in the MSKHC. A flow-chart of inclusion of study participants and follow-up rates is presented in Figure 1. Only participants responding to at least one

For peer review only

follow-up could be included in the analytic sample for the main analysis, giving a total of 2200 participants in the MHC, and 2520 in the MSKHC.

In the MHC, the mean age was 25 years and 57% were females. Seven percent had been exposed to discrimination, 47% experienced a high study pace, 19% experienced low social cohesion and 13% perceived the physical study environment as poor (Table 1). In the MSKHC, the mean age was 24.5 years and 59% were female. Eight percent had been exposed to discrimination, 55% experienced a high study pace, 24% experienced low social cohesion and 15% perceived the physical study environment as poor (Table 1). Characteristics of all study participants stratified by exposure status is presented in Table 2 and 3.

Table 1. Characteristics of the participants in the two cohorts

	**Mental health cohort (n = 2503)**	**Musculoskeletal health cohort (n = 2889)**
Age, mean (SD)	25.0 (6.4)	24.5 (6.1)
Gender, n (%)		
Female	1427 (57%)	1699 (59%)
Male	1065 (43%)	1173 (41%)
Other	11 (0%)	17 (1%)
Living Status, n (%)		
Alone	695 (28%)	818 (28%)
With others	1808 (72%)	2071 (72%)
Education type, n (%)		
Medical/Health	1184 (47%)	1288 (47%)
Technical	989 (40%)	1236 (43%)
Social sciences/humanities	228 (9%)	238 (8%)
Economic	59 (2%)	87 (3%)
Other	43 (2%)	40 (1%)
Highest parental education, n (%)		
University	1826 (73%)	2110 (73%)
Below university	677 (27%)	779 (27%)
Study year, n (%)		
First Year	1032 (41%)	1216 (42%)
Above First Year	1471 (59 %)	1673 (58%)
Place of birth, n (%)		
Sweden	1995 (80%)	2261 (78%)
Scandinavia	90 (4%)	97 (3%)
Europe	151 (6%)	174 (6%)

For peer review only

Outside Europe	267 (11%)	357 (12%)
Discrimination, n (%)	174 (7%)	241 (8%)
High study pace, n (%)	1180 (47%)	1592 (55%)
Low social cohesion, n (%)	479 (19%)	689 (24 %)
Poor physical study environment, n (%)	327 (13%)	438 (15%)

Table 2. Characteristics of the participants in the mental health cohort by exposure status.

	**Discrimination**		**High study pace**		**Low social cohesion ***		**Poor physical environment**	
	Unexposed (n=2329)	***Exposed (n=174)***	***Unexposed (n=1323)***	***Exposed (n=1180)***	***Unexposed (n=1986)***	***Exposed (n=479)***	***Unexposed (n=2176)***	***Exposed (n=327)***
Age, mean (SD)	24.9 (6.3)	25.9 (7.1)	25.2 (6.5)	24.8 (6.4)	24.7 (6.2)	25.4 (6.6)	24.8 (6.3)	26.5 (7.1)
Gender, n (%)								
Female	1301 (56%)	126 (72%)	680 (51%)	747 (63%)	1145 (58%)	254 (53%)	1231 (57%)	196 (60%)
Male	1019 (44%)	46 (26%)	639 (48%)	426 (36%)	834 (42%)	221 (46%)	936 (43%)	129 (39%)
Other**	-	-	-	-	-	-	-	-
Living Status, n (%)								
Alone	637 (27%)	58 (33%)	363 (27%)	332 (28%)	549 (28%)	140 (29%)	605 (28%)	90 (28%)
With others	1692 (73%)	116 (67%)	960 (73%)	848 (72%)	1437 (72%)	339 (71%)	1571 (72%)	237 (72%)
Education program, n (%)								
Medical/Health	1104 (47%)	80 (46%)	708 (54%)	476 (40%)	976 (49%)	189 (40%)	1019 (47%)	165 (51%)
Technical	926 (40%)	63 (36%)	451 (34%)	538 (46%)	774 (39%)	202 (42%)	891 (41%)	98 (30%)
Social sciences/ humanities	205 (9%)	23 (13%)	105 (8%)	123 (10%)	148 (8%)	74 (15%)	193 (9%)	35 (11%)
Economic**	-	-	-	-	-	-	-	-
Other**	-	-	-	-	-	-	-	-
Highest parental education, n (%)								
University	1706 (73%)	120 (69%)	967 (73%)	859 (73%)	1479 (74%)	324 (68%)	1592 (73%)	234 (72%)
Below university	623 (27%)	54 (31%)	356 (27%)	321 (27%)	507 (26%)	155 (32%)	584 (27%)	93 (28%)
Study year, n (%)								
First Year	984 (42%)	48 (28%)	507 (38%)	525 (44%)	840 (42%)	172 (36%)	919 (42%)	113 (35%)
Above First Year	1345 (58%)	126 (72%)	816 (62%)	655 (66%)	1146 (58%)	307 (64%)	1257 (58%)	214 (65%)
Place of birth, n (%)								
Sweden	1873 (80%)	122 (70%)	1050 (79%)	945 (80%)	1599 (81%)	371 (78%)	1721 (79%)	274 (84%)
Scandinavia**	-	-	52 (4%)	38 (3%)	77 (4%)	12 (3%)	-	-
Europe	-	-	83 (6%)	68 (6%)	108 (5%)	37 (8%)	-	-
Outside Europe	229 (10%)	38 (22%)	138 (10%)	129 (11%)	202 (10%)	59 (12%)	231 (11%)	36 (11%)

*38 persons excluded due to studying at programs not organized in study groups
**Some numbers are not presented due to low cell counts

For peer review only

Table 3. Characteristics of the participants in the musculoskeletal health cohort by exposure status.

	Discrimination		High study pace		Low social Cohesion *		Poor physical environment	
	Unexposed (n=2648)	*Exposed (n=241)*	*Unexposed (n=1297)*	*Exposed (n=1592)*	*Unexposed (n=2152)*	*Exposed (n=689)*	*Unexposed (n=2451)*	*Exposed (n=438)*
Age, mean (SD)	24.5 (6.1)	24.7 (6.1)	24.8 (6.2)	24.3 (6.0)	24.4 (6.0)	24.6 (6.0)	24.3 (5.9)	25.6 (6.8)
Gender, n (%)								
Female	1537 (58%)	162 (67%)	674 (52%)	1025 (64%)	1289 (60%)	379 (55%)	1428 (58%)	271 (62%)
Male	1098 (41%)	75 (31%)	616 (47%)	557 (35%)	852 (40%)	304 (44%)	1012 (41%)	161 (37%)
Other**	-	-	-	-	-	-	-	-
Living Status, n (%)								
Alone	745 (28%)	73 (30%)	353 (27%)	465 (29%)	610 (28%)	202 (29%)	697 (28%)	121 (28%)
With others	1903 (72%)	168 (70%)	944 (73%)	1127 (71%)	1542 (72%)	487 (71%)	1754 (72%)	317 (72%)
Education program, n (%)								
Medical/Health	1191 (45%)	97 (40%)	681 (53%)	607 (38%)	1017 (47.3)	253 (37%)	1088 (44%)	200 (46%)
Technical	1133 (43%)	103 (43%)	461 (36%)	775 (49%)	874 (40.6)	336 (49%)	1086 (44%)	150 (34%)
Social sciences/ humanities	209 (8%)	29 (12%)	96 (7%)	142 (9%)	152 (7%)	82 (12%)	192 (8%)	46 (11%)
Economic**	-	-	32 (3%)	55 (4%)	-	-	-	-
Other**	-	-	27 (2%)	13 (1%)	-	-	-	-
Highest parental education, n (%)								
University	1946 (73%)	164 (68%)	956 (74%)	1154 (72%)	1603 (74%)	476 (69%)	1793 (73%)	317 (72%)
Below university	702 (27%)	77 (32%)	341 (26%)	438 (28%)	549 (26%)	213 (31%)	658 (27%)	121 (28%)
Study year, n (%)								
First Year	1131 (43%)	85 (35%)	501 (39%)	715 (45%)	943 (44%)	249 (36%)	1054 (43%)	162 (37%)
Above First Year	1517 (57%)	156 (65%)	796 (61%)	877 (55%)	1209 (56%)	440 (64%)	1397 (57%)	276 (63%)
Place of birth, n (%)								
Sweden	2097 (79%)	164 (68%)	1021 (79%)	1240 (78%)	1707 (79%)	522 (76%)	1903 (78%)	358 (82%)
Scandinavia**	-	-	48 (4%)	49 (3%)	78 (4%)	18 (3%)	-	-
Europe**	-	-	81 (6%)	93 (6%)	119 (6%)	50 (7%)	-	-
Outside Europe	301 (11%)	56 (23%)	147 (11%)	210 (13%)	248 (12%)	99 (14%)	304 (12%)	53 (12%)

*48 persons excluded due to studying at programs not organized in study groups
** Some numbers are not presented due to low cell count

Risk analysis

The number of outcome events at each time-period is presented in Table 4. When comparing the hazard of the outcomes between exposed and unexposed participants, discrimination gave adjusted HRs of 1.75 (95% CI: 1.40 – 2.19) for moderate or worse mental health problems and 1.34 (95% CI:

1.08 – 1.67) for disabling pain in any bodily location. High study pace gave adjusted HRs of 1.70 (95% CI: 1.48 – 1.94) for mental health problems, and 1.24 (95% CI: 1.09 – 1.42) for disabling pain. Low social cohesion gave adjusted HRs of 1.51 (95% CI: 1.29 – 1.77) for mental health problems and 1.09 (95% CI: 0.93 – 1.26) for disabling pain. Poor physical environment gave adjusted HRs of 1.20 (95% CI: 0.99 – 1.45) for mental health problems, and 1.23 (95% CI: 1.04 – 1.47) for disabling pain (Table 5).

Table 4. Number of events and participants at risk at each time-period

	Mental health cohort			Musculoskeletal health cohort		
Time-period	*Events (n)*	*At risk (n)**	*Hazard**	*Events (n)*	*At risk (n)**	*Hazard**
0-3 months	502	2087	0.24	474	2366	0.20
3-6 months	180	1436	0.13	227	1713	0.13
6-9 months	133	1163	0.11	140	1368	0.10
9-12 months	94	997	0.09	115	1193	0.10

*Number are not equal to the analytic sample since not all participants were observed at all time-points.
**The hazard equals the probability of the event (Events / Events + At risk) conditional on not having had the event earlier.

Table 5. Hazard ratios of mental health problems and disabling pain in any bodily location comparing exposed to unexposed for different aspects of a poor study environment.

	Mental health problems		Disabling musculoskeletal pain	
	Crude HR (95% CI)	*Adjusted HR* (95% CI)*	*Crude HR (95% CI)*	*Adjusted HR* (95% CI)*
Discrimination	1.89 (1.52 – 2.35)	1.75 (1.40 – 2.19)	1.38 (1.12 – 1.71)	1.34 (1.08 – 1.67)
High study pace	1.83 (1.61 – 2.09)	1.70 (1.48 – 1.94)	1.21 (1.06 – 1.38)	1.24 (1.09 – 1.42)
Low social cohesion	1.55 (1.33 – 1.80)	1.51 (1.29 – 1.77)	1.07 (0.92 – 1.24)	1.09 (0.93 – 1.26)
Poor physical environment	1.17 (0.97 – 1.41)	1.20 (0.99 – 1.45)	1.26 (1.06 – 1.49)	1.23 (1.04 – 1.47)

*Adjusted for gender, age, living situation, education type, year of study, place of birth and highest level of parental education.

Sensitivity analyses

Additionally adjusting for baseline levels of depression, anxiety, and stress symptoms gave the following HRs for mental health problems: Discrimination (HR: 1.34 [95% CI: 1.07 - 1.69]); High study pace (HR: 1.18 [95% CI: 1.02 - 1.36]); Low social cohesion (HR: 1.28 [95% CI: 1.09 - 1.50]): Poor physical environment (HR: 1.02 [0.84 - 1.24]). For the outcome disabling pain, the HRs adjusted for

For peer review only

baseline levels of depression, anxiety, and stress symptoms were: Discrimination (HR: 1.24 [95% CI: 0.99-1.54]); High study pace (HR: 1.13 [95% CI: 0.98-1.30]); Low social cohesion (HR: 1.02 [95% CI: 0.87-1.19]); Poor physical environment (HR: 1.18 [95% CI: 0.99-1.41]).

Discussion

Several aspects of the study environment were associated with the incidence of mental health problems and disabling musculoskeletal pain in this sample of Swedish university students, even after adjustment for multiple potential confounders. Discrimination at university was experienced by 7-8% of our sample and associated with the incidence of both mental health problems and disabling pain. Our results are in line with previous findings on discrimination and mental health (16, 17) but highlights that discrimination in the university setting may also be of importance in relation to pain. The association was somewhat weaker for disabling pain than for mental health, which is in line with earlier research suggesting that discrimination increases the risk of disabling pain by mediation from psychological distress (18).

High study pace was commonly experienced in our sample and associated with the incidence of both mental health problems and disabling pain, which corroborates earlier research (19, 20). High study pace was, like discrimination, more strongly associated with the incidence of mental health problems than with disabling pain. Low social cohesion at university was experienced by about a fifth of our sample and associated with higher incidence of mental health problems. This association has not, to our knowledge, been studied among university students previously, but the results are in line with research showing associations between mental health problems and lack of school cohesion among adolescents (23). Low social cohesion was also associated with higher incidence of disabling pain, although the association was weak, and the confidence intervals did not exclude an effect in the opposite direction. Although inconclusive, these results are in line with earlier research from working populations (24). The physical environment at university was perceived as good by the majority of our sample. However, perceiving the environment as poor was associated with both incidence of

For peer review only

mental health problems and disabling pain, although for mental health problems the confidence intervals did not exclude an association in the opposite direction. These findings support earlier findings of association between physical aspects of the study environment and pain (25, 26).

Mental health problems and musculoskeletal pain are some of the most pressing public health concerns for young adults (1) and are highly prevalent among university students (2-4). Our results indicate that aspects of the study environment may be an important target for universities trying to tackle the high levels of mental health problems and disabling pain among university students. Addressing poor study environment issues might to some extent reduce incidence of mental health problems and disabling pain among university students. Future research on the effect of intervening on these study environment factors on mental health and pain is warranted.

Strengths and limitations

This study has several strengths but also limitations. We were able to recruit a large sample of university students, which allowed us to study the associations between different study environment exposures and the outcomes with good precision. Further, the longitudinal design and the control for multiple potential confounders strengthens the level of evidence for a causal association.

There are, however, limitations hindering us from ascertaining causality. First, there is still the possibly unmeasured and residual confounding. Factors such as genetic vulnerability and neuroticism may have affected both the exposures and the outcome and thereby confounded our estimates. We assumed that subclinical symptoms at baseline were on the causal pathway from the exposure to the outcomes. If this assumption does not hold, we have the risk of reverse causality. Our sensitivity analysis showed that all associations weakened when controlling for baseline levels of depression, anxiety and stress symptoms, as expected both if subclinical symptoms were mediators or confounders. However, all exposures, except poor physical environment, were still associated with the incidence of mental health problems, suggesting that reverse causality is unlikely to explain away the findings. For the incidence of disabling pain, the confidence intervals of all exposures included

For peer review only

the null when controlling for baseline mental health symptoms, leaving these associations more vulnerable to confounding by prior mental health problems. Second, since there was loss to follow-up in this study, our estimates may be affected by selection bias, especially if unmeasured risk factors of the outcomes were also associated with loss to follow-up. Third, the outcomes were measured using valid questionnaires, which limits the risk for misclassification in the outcomes. The exposures were measured using single item questions, and it is possible that misclassification of the exposures is present. We believe that this error is most likely to be non-differential, which would attenuate the true associations. However, since self-rated measures were used, the risk of differential misclassification cannot be ruled out. The use of single item exposures also limits our ability to differentiate between different aspects of the exposures. For instance, our measure of the perceived physical environment measure is quite crude and does not differentiate between different aspects of the physical environment (e.g. ergonomic workspaces, lighting, noise etc.).

Lastly, our sample was restricted to certain universities, and to participants agreeing to participate, and does not fully represent the overall Swedish student population. Thus, it is unclear how well these results may generalize to other populations. We believe, however, that found associations found in this study are similar to those in other populations, a belief supported by the alignment of our results to those of prior research.

Conclusions

Several aspects of the study environment were associated with the incidence of mental health problems and disabling musculoskeletal pain in our sample of Swedish university students, after controlling for multiple potential confounders.

For peer review only

Figure 1. Flowchart of the inclusion of participants. MHC = Mental health cohort; MSKHC = Musculoskeletal health cohort.

Acknowledgements

We thank Pierre Côté, Klara Edlund and Tobias Sundberg for their important contributions in planning and performing the SUN-study data collection. We would also like to express our appreciation to all the participating students for their contribution to the SUN-study.

Funding

This research project was funded by the Swedish Research Council for Health, Working Life and Welfare (FORTE), grant number FORTE2018-00402. The SUN-study also received financial support from the Public Health Agency of Sweden.

For peer review only

Competing interests

The authors report no competing interests.

Contributorship statement

Conceptualization – Fred Johansson, Jessica Billquist, Hanna Andreasson, and Eva Skillgate

Data curation – Fred Johansson

Formal Analysis – Fred Johansson

Funding acquisition – Eva Skillgate

Investigation – Fred Johansson, Clara Onell, and Eva Skillgate

Methodology – Fred Johansson and Eva Skillgate

Project administration – Eva Skillgate

Supervision – Anne H Berman and Eva Skillgate

Writing – original draft – Fred Johansson, Jessica Billquist, Hanna Andreasson and Eva Skillgate

Writing – review & editing – Fred Johansson, Jessica Billquist, Hanna Andreasson, Irene Jensen, Clara Onell, Anne H Berman, and Eva Skillgate

Data Availability Statement

The dataset analyzed in the current study is not publicly available due to secondary confidentiality and privacy of the participants.

Ethics Approval

The study was approved by the Swedish Ethical Review Authority (2019-03276), and all participants provided informed consent electronically before entering the study.

References

1. Mokdad AH, Forouzanfar MH, Daoud F, Mokdad AA, El Bcheraoui C, Moradi-Lakeh M, et al. Global burden of diseases, injuries, and risk factors for young people's health during 1990-2013: a systematic analysis for the Global Burden of Disease Study 2013. Lancet. 2016;387(10036):2383-401.
2. Rotenstein LS, Ramos MA, Torre M, Segal JB, Peluso MJ, Guille C, et al. Prevalence of Depression, Depressive Symptoms, and Suicidal Ideation Among Medical Students: A Systematic Review and Meta-Analysis. JAMA: Journal of the American Medical Association. 2016;316(21):2214-36.
3. Ibrahim AK, Kelly SJ, Adams CE, Glazebrook C. A systematic review of studies of depression prevalence in university students. J Psychiatr Res. 2013;47(3):391-400.
4. Sheldon E, Simmonds-Buckley M, Bone C, Mascarenhas T, Chan N, Wincott M, et al. Prevalence and risk factors for mental health problems in university undergraduate students: A systematic review with meta-analysis. J Affect Disord. 2021;287:282-92.
5. Auerbach RP, Alonso J, Axinn WG, Cuijpers P, Ebert DD, Green JG, et al. Mental disorders among college students in the World Health Organization World Mental Health Surveys. Psychol Med. 2016;46(14):2955-70.
6. Keyes CL, Eisenberg D, Perry GS, Dube SR, Kroenke K, Dhingra SS. The relationship of level of positive mental health with current mental disorders in predicting suicidal behavior and academic impairment in college students. J Am Coll Health. 2012;60(2):126-33.
7. Rudman A, Gustavsson JP. Burnout during nursing education predicts lower occupational preparedness and future clinical performance: a longitudinal study. Int J Nurs Stud. 2012;49(8):988-1001.
8. Kim HJ, Boo S, Meeker TJ. Pain Prevalence, Management and Interference Among University Students in South Korea: An Exploratory Cross-Sectional Study. J Pain Res. 2021;14:2423-31.
9. Robertson D, Kumbhare D, Nolet P, Srbely J, Newton G. Associations between low back pain and depression and somatization in a Canadian emerging adult population. J Can Chiropr Assoc. 2017;61(2):96-105.

For peer review only

10. El Ansari W, Stock C, Snelgrove TUSHGS, Hu X, Parke S, Davies S, et al. Feeling Healthy? A Survey of Physical and Psychological Wellbeing of Students from Seven Universities in the UK. Int J Environ Res Public Health. 2011;8(5):1308-23.
11. Serbic D, Friedrich C, Murray R. Psychological, social and academic functioning in university students with chronic pain: A systematic review. J Am Coll Health. 2021:1-15.
12. Bair MJ, Robinson RL, Katon W, Kroenke K. Depression and Pain Comorbidity: A Literature Review. Arch Intern Med. 2003;163(20):2433-45.
13. Kroenke K, Wu J, Bair MJ, Krebs EE, Damush TM, Tu W. Reciprocal relationship between pain and depression: a 12-month longitudinal analysis in primary care. J Pain. 2011;12(9):964-73.
14. Bondesson E, Larrosa Pardo F, Stigmar K, Ringqvist Å, Petersson IF, Jöud A, et al. Comorbidity between pain and mental illness - Evidence of a bidirectional relationship. Eur J Pain. 2018;22(7):1304-11.
15. Gröjer A, Wikström Öbrand I, Elenäs J, Gillström P, Dryler H, Eriksson G. Studentspegeln 2016. 2017.
16. Pascoe EA, Smart Richman L. Perceived discrimination and health: a meta-analytic review. Psychol Bull. 2009;135(4):531-54.
17. Schmitt MT, Branscombe NR, Postmes T, Garcia A. The consequences of perceived discrimination for psychological well-being: a meta-analytic review. Psychol Bull. 2014;140(4):921-48.
18. Brown TT, Partanen J, Chuong L, Villaverde V, Chantal Griffin A, Mendelson A. Discrimination hurts: The effect of discrimination on the development of chronic pain. Soc Sci Med. 2018;204:1-8.
19. Barker ET, Howard AL, Villemaire-Krajden R, Galambos NL. The Rise and Fall of Depressive Symptoms and Academic Stress in Two Samples of University Students. J Youth Adolesc. 2018;47(6):1252-66.
20. Nerdrum P, Rust∅en T, Helge R∅nnestad M. Psychological Distress Among Nursing, Physiotherapy and Occupational Therapy Students: A Longitudinal and Predictive Study. Scandinavian Journal of Educational Research. 2009;53(4):363-78.
21. Grimby-Ekman A, Andersson EM, Hagberg M. Analyzing musculoskeletal neck pain, measured as present pain and periods of pain, with three different regression models: a cohort study. BMC Musculoskelet Disord. 2009;10:73.
22. Leigh-Hunt N, Bagguley D, Bash K, Turner V, Turnbull S, Valtorta N, et al. An overview of systematic reviews on the public health consequences of social isolation and loneliness. Public Health. 2017;152:157-71.
23. Santini ZI, Pisinger VSC, Nielsen L, Madsen KR, Nelausen MK, Koyanagi A, et al. Social Disconnectedness, Loneliness, and Mental Health Among Adolescents in Danish High Schools: A Nationwide Cross-Sectional Study. Front Behav Neurosci. 2021;15:632906.
24. Hoogendoorn WE, van Poppel MN, Bongers PM, Koes BW, Bouter LM. Systematic review of psychosocial factors at work and private life as risk factors for back pain. Spine (Phila Pa 1976). 2000;25(16):2114-25.
25. Ogunlana MO, Govender P, Oyewole OO. Prevalence and patterns of musculoskeletal pain among undergraduate students of occupational therapy and physiotherapy in a South African university. Hong Kong Physiother J. 2021;41(1):35-43.
26. Gheysvandi E, Dianat I, Heidarimoghadam R, Tapak L, Karimi-Shahanjarini A, Rezapur-Shahkolai F. Neck and shoulder pain among elementary school students: prevalence and its risk factors. BMC Public Health. 2019;19(1):1299.
27. Edlund K, Sundberg T, Johansson F, Onell C, Rudman A, Holm LW, et al. Sustainable UNiversity Life (SUN) study: protocol for a prospective cohort study of modifiable risk and prognostic factors for mental health problems and musculoskeletal pain among university students. BMJ Open. 2022;12(4):e056489.
28. Henry JD, Crawford JR. The short-form version of the Depression Anxiety Stress Scales (DASS-21): construct validity and normative data in a large non-clinical sample. Br J Clin Psychol. 2005;44(Pt 2):227-39.

For peer review only

29. Alfonsson S, Wallin E, Maathz P. Factor structure and validity of the Depression, Anxiety and Stress Scale-21 in Swedish translation. J Psychiatr Ment Health Nurs. 2017;24(2-3):154-62.
30. Kuorinka I, Jonsson B, Kilbom A, Vinterberg H, Biering-Sørensen F, Andersson G, et al. Standardised Nordic questionnaires for the analysis of musculoskeletal symptoms. Appl Ergon. 1987;18(3):233-7.
31. Hefner J, Eisenberg D. Social Support and Mental Health Among College Students. Am J Orthopsychiatry. 2009;79(4):491-9.
32. Bewick B, Koutsopoulou G, Miles J, Slaa E, Barkham M. Changes in undergraduate students' psychological well-being as they progress through university. Studies in Higher Education. 2010;35(6):633-45.
33. Beiter R, Nash R, McCrady M, Rhoades D, Linscomb M, Clarahan M, et al. The prevalence and correlates of depression, anxiety, and stress in a sample of college students. J Affect Disord. 2015;173:90-6.
34. Hernan MA, Robins JM. Causal Inference: What If. 1st ed. Boca Raton: Chapman & Hall/CRC; 2020.
35. Allison PD. Discrete-Time Methods for the Analysis of Event Histories. Sociol Methodol. 1982;13:61-98.

For peer review only

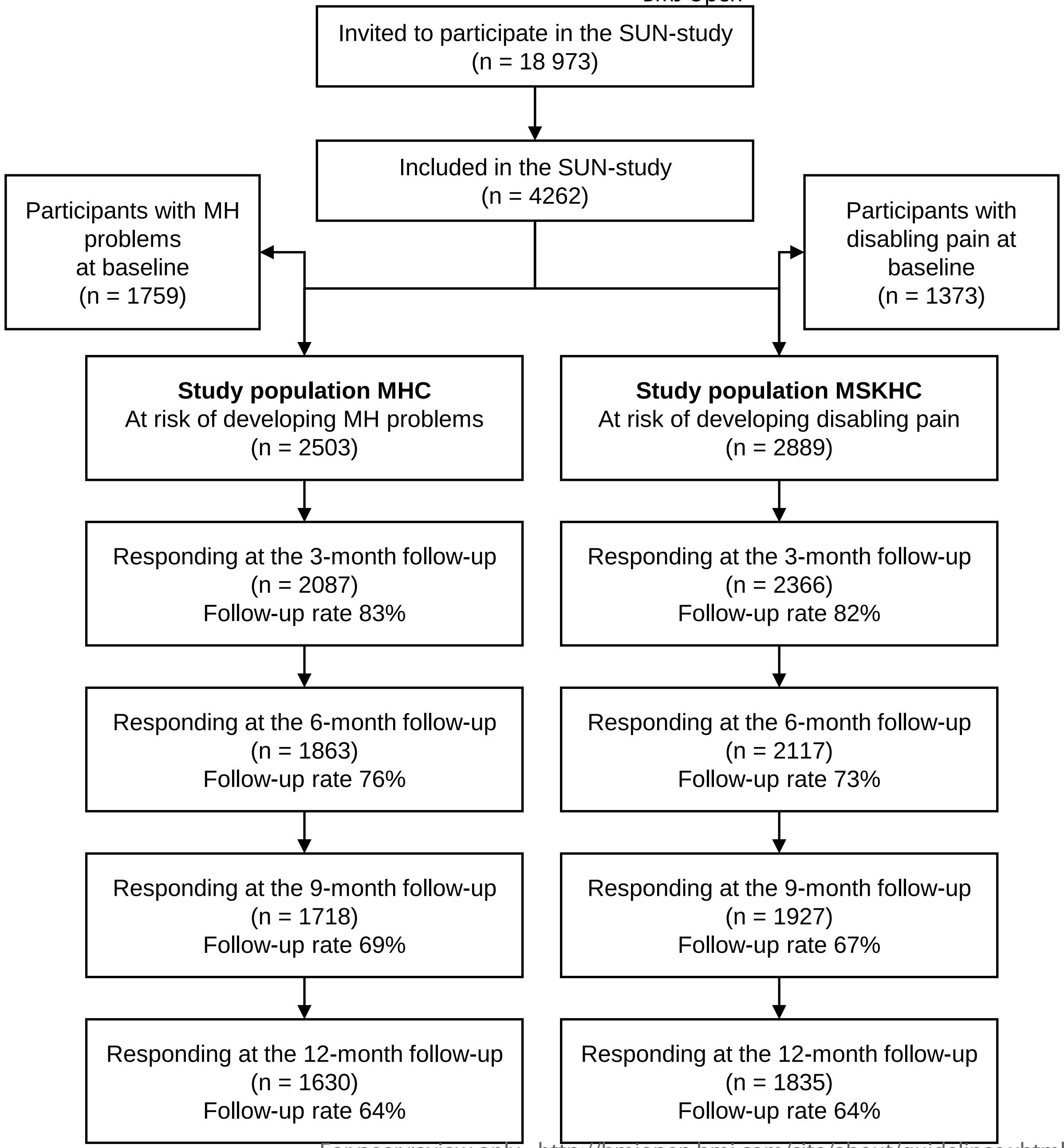

For peer review only - http://bmjopen.bmj.com/site/about/guidelines.xhtml

Figure 1. Flowchart of the inclusion of participants. MHC = Mental health cohort; MSKHC = Musculoskeletal health cohort.

STROBE Statement—Checklist of items that should be included in reports of ***cohort studies***

	Item No	Recommendation	Page No
**Title and abstract**	1	(*a*) Indicate the study's design with a commonly used term in the title or the abstract<br>(*b*) Provide in the abstract an informative and balanced summary of what was done and what was found	1-2
**Introduction**			
Background/rationale	2	Explain the scientific background and rationale for the investigation being reported	4-5
Objectives	3	State specific objectives, including any prespecified hypotheses	5
**Methods**			
Study design	4	Present key elements of study design early in the paper	6
Setting	5	Describe the setting, locations, and relevant dates, including periods of recruitment, exposure, follow-up, and data collection	6
Participants	6	(*a*) Give the eligibility criteria, and the sources and methods of selection of participants. Describe methods of follow-up<br>(*b*) For matched studies, give matching criteria and number of exposed and unexposed	6
Variables	7	Clearly define all outcomes, exposures, predictors, potential confounders, and effect modifiers. Give diagnostic criteria, if applicable	6-8
Data sources/ measurement	8*	For each variable of interest, give sources of data and details of methods of assessment (measurement). Describe comparability of assessment methods if there is more than one group	6-8
Bias	9	Describe any efforts to address potential sources of bias	9
Study size	10	Explain how the study size was arrived at	-
Quantitative variables	11	Explain how quantitative variables were handled in the analyses. If applicable, describe which groupings were chosen and why	9
Statistical methods	12	(*a*) Describe all statistical methods, including those used to control for confounding<br>(*b*) Describe any methods used to examine subgroups and interactions<br>(*c*) Explain how missing data were addressed<br>(*d*) If applicable, explain how loss to follow-up was addressed<br>(*e*) Describe any sensitivity analyses	9
**Results**			
Participants	13*	(a) Report numbers of individuals at each stage of study—eg numbers potentially eligible, examined for eligibility, confirmed eligible, included in the study, completing follow-up, and analysed<br>(b) Give reasons for non-participation at each stage<br>(c) Consider use of a flow diagram	10 and figure 1
Descriptive data	14*	(a) Give characteristics of study participants (eg demographic, clinical, social) and information on exposures and potential confounders<br>(b) Indicate number of participants with missing data for each variable of interest<br>(c) Summarise follow-up time (eg, average and total amount)	10, table 1-4
Outcome data	15*	Report numbers of outcome events or summary measures over time	Table4

For peer review only

For peer review only - http://bmjopen.bmj.com/site/about/guidelines.xhtml

Main results	16	(*a*) Give unadjusted estimates and, if applicable, confounder-adjusted estimates and their precision (eg, 95% confidence interval). Make clear which confounders were adjusted for and why they were included<br>(*b*) Report category boundaries when continuous variables were categorized<br>(*c*) If relevant, consider translating estimates of relative risk into absolute risk for a meaningful time period	Table 4 and 5
Other analyses	17	Report other analyses done—eg analyses of subgroups and interactions, and sensitivity analyses	11
**Discussion**			
Key results	18	Summarise key results with reference to study objectives	11
Limitations	19	Discuss limitations of the study, taking into account sources of potential bias or imprecision. Discuss both direction and magnitude of any potential bias	12-13
Interpretation	20	Give a cautious overall interpretation of results considering objectives, limitations, multiplicity of analyses, results from similar studies, and other relevant evidence	14
Generalisability	21	Discuss the generalisability (external validity) of the study results	14
**Other information**			
Funding	22	Give the source of funding and the role of the funders for the present study and, if applicable, for the original study on which the present article is based	19

*Give information separately for exposed and unexposed groups.

Note: An Explanation and Elaboration article discusses each checklist item and gives methodological background and published examples of transparent reporting. The STROBE checklist is best used in conjunction with this article (freely available on the Web sites of PLoS Medicine at http://www.plosmedicine.org/, Annals of Internal Medicine at http://www.annals.org/, and Epidemiology at http://www.epidem.com/). Information on the STROBE Initiative is available at http://www.strobe-statement.org.

For peer review only

For peer review only - http://bmjopen.bmj.com/site/about/guidelines.xhtml

BMJ Open

Study Environment and the Incidence of Mental Health Problems and Activity-Limiting Musculoskeletal Problems among University Students: The SUN Cohort Study

Journal:	*BMJ Open*
Manuscript ID	bmjopen-2023-072178.R1
Article Type:	Original research
Date Submitted by the Author:	29-Jun-2023
Complete List of Authors:	Johansson, Fred; Sophiahemmet University, Department of Health Promotion Science<br>Billquist, Jessica; Uppsala Universitet, Department of Psychology<br>Andreasson, Hanna; Uppsala Universitet, Department of Psychology<br>Jensen, Irene; Karolinska Institutet, Institute of Environmental Medicine<br>Onell, Clara; Sophiahemmet University, Department of Health Promotion Science<br>Berman, Anne H.; Uppsala Universitet, Department of Psychology; Karolinska Institutet, Department of Clinical Neuroscience<br>Skillgate, Eva; Sophiahemmet University, Department of Health Promotion Science; Karolinska Institutet, Institute of Environmental Medicine
**Primary Subject Heading**:	Epidemiology
Secondary Subject Heading:	Mental health
Keywords:	MENTAL HEALTH, Depression & mood disorders < PSYCHIATRY, Musculoskeletal disorders < ORTHOPAEDIC & TRAUMA SURGERY, EPIDEMIOLOGY

SCHOLARONE™
Manuscripts

I, the Submitting Author has the right to grant and does grant on behalf of all authors of the Work (as defined in the below author licence), an exclusive licence and/or a non-exclusive licence for contributions from authors who are: i) UK Crown employees; ii) where BMJ has agreed a CC-BY licence shall apply, and/or iii) in accordance with the terms applicable for US Federal Government officers or employees acting as part of their official duties; on a worldwide, perpetual, irrevocable, royalty-free basis to BMJ Publishing Group Ltd ("BMJ") its licensees and where the relevant Journal is co-owned by BMJ to the co-owners of the Journal, to publish the Work in this journal and any other BMJ products and to exploit all rights, as set out in our licence.

The Submitting Author accepts and understands that any supply made under these terms is made by BMJ to the Submitting Author unless you are acting as an employee on behalf of your employer or a postgraduate student of an affiliated institution which is paying any applicable article publishing charge ("APC") for Open Access articles. Where the Submitting Author wishes to make the Work available on an Open Access basis (and intends to pay the relevant APC), the terms of reuse of such Open Access shall be governed by a Creative Commons licence – details of these licences and which Creative Commons licence will apply to this Work are set out in our licence referred to above.

Other than as permitted in any relevant BMJ Author's Self Archiving Policies, I confirm this Work has not been accepted for publication elsewhere, is not being considered for publication elsewhere and does not duplicate material already published. I confirm all authors consent to publication of this Work and authorise the granting of this licence.

For peer review only

Study Environment and the Incidence of Mental Health Problems and Activity-Limiting Musculoskeletal Problems among University Students: The SUN Cohort Study

Authors

Fred Johansson [1*]

Jessica Billquist[2]

Hanna Andreasson[2]

Irene Jensen[3]

Clara Onell [1]

Anne H Berman[2,4]

Eva Skillgate [1,3,5]

[1]Department of Health Promotion Science, Sophiahemmet University, Stockholm, Sweden

[2]Department of Psychology, Uppsala University, Uppsala, Sweden

[3]Unit of Intervention and Implementation Research for Worker Health, Institute of Environmental Medicine, Karolinska Institutet, Stockholm, Sweden

[4]Department of Clinical Neuroscience, Karolinska Institutet, Stockholm, Sweden

[5]Naprapathögskolan – Scandinavian College of Naprapathic Manual Medicine, Stockholm, Sweden

* Corresponding author: Fred Johansson, Sophiahemmet Högskola, Box 5605, 114 86 Stockholm, Sweden. Email: fred.johansson@shh.se. Phone: +46739516158

For peer review only

Word count: 3827

Keywords: Students; Mental Health; Musculoskeletal Problems; Perceived Discrimination; Social Cohesion; Stress, Psychological

Abstract

Objective. To determine the association between different aspects of study environment and the incidence of mental health problems and activity-limiting musculoskeletal problems. **Design, setting and participants.** We recruited a cohort of 4262 Swedish university students of whom 2503 (59%) were without moderate or worse mental health problems and 2871 (67%) without activity-limiting musculoskeletal problems at baseline. The participants were followed at five time-points over on year using web surveys. **Exposures.** Self-rated discrimination, high study pace, low social cohesion and poor physical environment measured at baseline. **Outcomes:** Self-rated mental health problems, defined as scoring above cut-off on any of the subscales of the Depression, anxiety, and stress scale. Self-rated activity-limiting musculoskeletal problems in any body location, assessed by the Nordic Musculoskeletal Questionnaire. **Statistical analysis.** Discrete survival-time analysis was used to estimate the hazard rate ratio (HR) of each exposure-outcome combination while adjusting for gender, age, living situation, education type, year of studies, place of birth and parental education as potential confounders. **Results.** For discrimination, adjusted HRs were 1.75 (95% CI: 1.40 – 2.19) for mental health problems and 1.39 (95% CI: 1.12 – 1.72) for activity-limiting musculoskeletal problems. For high study pace, adjusted HRs were 1.70 (95% CI: 1.48 – 1.94) for mental health problems and 1.25 (95% CI: 1.09 – 1.43) for activity-limiting musculoskeletal problems. For low social cohesion, adjusted HRs were 1.51 (95% CI: 1.29 – 1.77) for mental health problems and 1.08 (95% CI: 0.93 – 1.25) for activity-limiting musculoskeletal problems. For perceived poor physical study environment, adjusted HRs were 1.20 (95% CI: 0.99 – 1.45) for mental health problems and 1.20 (95% CI: 1.01 – 1.43) for activity-limiting musculoskeletal problems. **Conclusions.** Several aspects of the study environment were associated with the incidence of mental health problems and activity-limiting musculoskeletal problems in this sample of Swedish university students.

For peer review only

Strengths and limitations of this study

- We recruited a large sample of students, which enables us to estimate associations between the exposures and the outcomes with good precision.
- We followed the participants over time, and were able to adjust for multiple potential confounders, which strengthens the evidence level for causal associations.
- There may still be residual and unmeasured confounding that could affect the associations.
- Exposures were measured with single items questions, which could increase the risk of misclassification.
- The sample was measured mostly during the COVID-19 pandemic and is not representative of Swedish university students overall, which may affect generalizability. We had some loss to follow-up, which could lead to selection bias that could affect the estimated associations.

For peer review only

Introduction

Mental health problems and musculoskeletal problems, such as neck and back pain, are leading causes of years lost due to disability in young people globally (1), and common among university students (2-5). Previous studies show that 35.7% of students from high-income countries meet criteria for at least one common mental disorder (5). Mental health problems are associated with increased risk of student attrition (5), academic impairment (6), reduced occupational preparedness and future work performance (7). Musculoskeletal pain is also common among university students (8-10) and associated with poorer academic functioning and lower quality of life (11).

Mental health problems and pain commonly co-occur (12). This comorbidity has been suggested to be characterized by bidirectional causation, with pain influencing mental health and vice versa (13, 14). Given the high comorbidity and bidirectional influences, it is likely that these conditions share many risk factors. Some risk factors are likely to affect students and non-students alike. Others, like the exposure to university study environments, are specific to students, and pose an important research target for understanding and preventing student health problems. Study environment is a multifaceted construct with several physical and psychosocial aspects. This study focuses on four specific aspects: discrimination, study pace, social cohesion, and physical study environment.

Seven percent of Swedish students report experiencing discrimination during their studies (15). Perceived discrimination is associated with mental and physical ill health (16) and is negatively associated with subsequent psychological well-being even after controlling for baseline levels of psychological well-being (17). Brown et al (18) showed that the association between discrimination and chronic pain was mediated through psychological distress in a general population sample. However, little is known as to how discrimination occurring specifically at the university affects students' health.

Perceived stress and academic demands have been associated with higher levels of depressive symptoms and psychological distress among students in longitudinal studies (19, 20), and perceived

For peer review only

work/study demands with neck and upper back pain among young adults (21). However, few studies have focused specifically on high study pace and its associations to mental health and activity-limiting musculoskeletal problems.

Social isolation and loneliness are well-established risk factors for mental health problems in general (22), but less is known about the impact of low social cohesion within the study environment. For adolescents, a lack of social cohesion at school and lack of classmate support are cross-sectionally associated with lower psychological well-being and higher symptom levels of depression and anxiety (23). Low workplace social support has also been identified as a risk factor for back pain among working adults (24), but to our knowledge there is no studies on the association between social cohesion and activity-limiting musculoskeletal problems among university students.

Physical aspects of the study environment such as repetitive movement, prolonged sitting and sitting position are associated with musculoskeletal pain among university students (25) and school children (26). However, there is a lack of research into how students' perceptions of physical study environment is associated with subsequent mental health problems and activity-limiting musculoskeletal problems.

In this study, we aimed to determine the associations between four aspects of study environment: discrimination; high study pace; low social cohesion; and perceived poor physical environment, and the incidence of mental health problems and activity-limiting musculoskeletal problems among Swedish university students.

Method

Design and participants

This study is based on the Sustainable UNiversity life (SUN) cohort (27), a longitudinal study aiming to identify risk and prognostic factors for mental health problems and musculoskeletal pain and problems in university students. Participants were recruited between August 2019 and December

For peer review only

2021, from eight universities in the greater Stockholm area and Örebro. The targeted universities constitute a convenience sample, selected to cover a variety of educational programs mainly within medicine, business, technology, and social sciences. Full-time undergraduate or graduate students (up to and including masters' level) at selected educational programs with at least one full year of academic studies left before graduation were eligible for inclusion.

Eligible students were informed about the study during an in-class presentation and/or by email with a link to the baseline survey. Students agreeing to participate were followed every three months for one year using web surveys. For the aim of studying incidence of mental health problems and activity-limiting musculoskeletal problems in this study, two cohorts were established: 1) the mental health cohort (MHC) including participants without moderate or more severe mental health problems at baseline; and 2) the musculoskeletal health cohort (MSKHC) including participants without activity-limiting musculoskeletal problems at any body location at baseline (Figure 1).

The study was approved by the Swedish Ethical Review Authority (2019-03276), and all participants provided informed consent electronically before entering the study. More information about the study methodology and data collection is available in the study protocol (27).

Patient and public involvement

We involved students by conducting focus group interviews on health challenges of university students during the planning phase of the study. We have also had an ongoing dialogue with student health services, educational offices and student unions during the planning and conduct of the study, as well as for dissemination of the results. Students and university officials have been involved in the selection of exposures and outcomes, in recruitment and data collection phase, and have been offered to take part of the results of the study (for instance by participating in seminars organized by the research group).

For peer review only

Measures

Outcomes

Mental health problems were measured with the short-form Depression Anxiety Stress Scale (DASS-21) (28), consisting of 21 items including subscales on depression, anxiety, and stress. Each subscale comprises seven items rated on a scale from 0 (Did not apply to me at all) to 3 (Applied to me very much, or most of the time), with a recall time of one week. The items were summed to give subscale-scores ranging from 0 to 21. Participants scoring above the cut-off for moderate symptoms levels on any of the scales ($\geq$ 7 on the depression subscale, $\geq$ 6 on the anxiety subscale, $\geq$ 10 on the stress subscale) were considered to have mental health problems. DASS-21 has shown good psychometric properties among Swedish university students (29).

Activity-limiting musculoskeletal problems was measured with the Nordic Musculoskeletal Questionnaire (NMQ) (30). Participants were asked if they *"at any time during the last three months have been prevented from doing your normal activities (e.g. work, studies, household activities, hobbies) due to trouble with:"* ankles/feet, knee, hip, lower back, upper back, shoulder, elbow, hand, and/or neck. The question was responded to dichotomously (Yes/No) for each body location. Participants responding Yes for any of the body locations were considered to have activity-limiting musculoskeletal problems.

Exposures

All exposures were measured at baseline.

Discrimination was measured by the question: *"Have you ever during the past year felt discriminated by other students, teachers or administrative personnel due to:"* over nine categories: gender, gender identity, gender expression, ethnicity, religion or beliefs, sexual orientation, disabilities, age, other [15]. Participants answering "Yes" on at least one of the categories were asked to rate how severely the discrimination had affected them on a scale from 0 (Not at all) to 10 (Worst imaginable way). Participants with ratings >3 were considered exposed to discrimination.

For peer review only

Study pace was measured by the questions: *"Do you feel that you are coping with the study pace?"* with three response categories: (1) *"I feel that I have energy and time to cope with the study pace",* (2) "*The study pace is high. I do my best, but I am afraid that it is not enough"* and (3) "*The study pace breaks me. We get more assignments than I have time for, and I feel that I cannot make it"*. Participant's choosing categories 2 or 3 were considered exposed to high study pace.

Social cohesion was measured by the question: *"How good is the cohesion with your course mates?"*, with four response alternatives: (1) *"The cohesion is pretty good. We help each other with the studies and there is always someone in the class to talk to*", (2) *"I do not think that our cohesion is good. We work independently, and it feels like it is not worth the inconvenience to try to change it*", (3) *"We do not have any cohesion, and no one takes any responsibility over our groupwork or the fellowshi*p" and (4) *"My education is not organized around study groups or classes"*. Participants choosing categories 2 or 3 were considered exposed to low social cohesion. Participants choosing the fourth category were excluded from the risk analyses.

Perceived physical environment was measured by asking: "*How do you in general experience the physical study environment at your university? (For example, public areas, lunchroom, lecture halls, toilets, outside environment around campus.)"* and rated on a scale from 0 (Very bad) to 10 (Very good). Participants with ratings of 0-5 were considered exposed to a poor physical study environment.

Covariates

Potential confounders were selected based on previous research (e.g. (4, 31-33)) and directed acyclic graphs (34). We included the covariates gender (female, male, other), age (continuous), living situation (alone, with others), education type (medical/health, technical, social sciences/humanities, economic, other), year of studies (first, second, above), place of birth (Sweden, Scandinavia, Europe, outside Europe) and highest level of parental education (university, below university) as a proxy for socioeconomic status.

For peer review only

Statistical analysis

We used discrete-time survival analysis to estimate associations between baseline exposures and the hazard of each of the outcomes. A participant was considered at risk for the outcome until the outcome was first registered or until censoring due to loss to follow-up or end of follow-up, whichever came first.

We used generalized linear models with binomial error distributions and complementary log-log links to estimate the hazard ratio (HR) of the outcomes comparing the exposed to the unexposed. Separate models were built for each exposure – outcome combination. This modeling approach is equivalent to Cox proportional hazard models when assuming that the underlying time variable is continuous (35). Time was measured as four time-periods (0 to 3 months, 3 to 6 months, 6 to 9 months, and 9 to 12 months). Plotting the log hazard over the four time-periods showed a fairly linear relationship for both outcomes, and therefore the time-period variable was treated as continuous. The proportional hazard assumption was evaluated by including interaction terms between time-period and the exposures and performing likelihood ratio tests comparing models with and without interaction terms. These indicated better fits for models assuming proportional hazards for all exposure – outcome combinations, except for the model estimating the association between discrimination and mental health problems.

Sensitivity analyses

Several additional sensitivity analyses were run as detailed below. First, subclinical symptom levels of mental health problems at baseline were assumed to be on the causal pathway and thus not included as covariates in the main analyses. To assess the sensitivity of our analyses to this assumption, we performed sensitivity analyses where the continuous scores of the three DASS-21 subscales measured at baseline were included as covariates. Second, data collection took place before and during the COVID-19 pandemic, which may have affected both our exposures and

For peer review only

outcomes thus potentially leading to confounding. About half of the participants entered the SUN-study before the COVID-19 pandemic started in Sweden on March 13, 2020 (45% in MHC and 41% in MSKHC), and almost all outcome data was collected after the onset of the pandemic (92% in the MHC and 83% in the MSKHC). To assess the robustness of our results to potential confounding by the COVID-19 pandemic we reran the main analyses while including a covariate indicating if the baseline survey, where exposures were measured, was collected before or after March 13, 2020 (Supplemental eTable 1). Third, to assess potential effect modification by gender, gender stratified risk analyses were performed (Supplemental eTable 2). Fourth, to assess potential selection bias we computed risk ratios (RR) of loss to follow-up at the 12-month follow-up between exposed and unexposed (Supplemental eTable 3) (36), and computed E-values defining the minimum association between missingness and the outcome needed to shift the point estimates of the main analysis to the null (37) (Supplemental eTable 4). Fifth, to investigate potential dose-response relationships for the exposures high study pace and low social cohesion we performed sensitivity analyses treating each response category as a separate exposure level (Supplemental eTable 5).

All analyses were performed using R version 4.1.2.

Results

We included 4262 participants, of whom 2503 (59%) were without moderate or worse mental health problems at baseline and were included in the MHC and 2871 (67%) were without activity-limiting musculoskeletal problems in any body location at baseline and included in the MSKHC. A flow-chart of inclusion of study participants and follow-up rates is presented in Figure 1. Only participants responding to at least one follow-up could be included in the analytic sample for the main analysis, giving a total of 2200 participants in the MHC, and 2513 in the MSKHC.

In the MHC, the mean age was 25 years and 57% were females. Seven percent had been exposed to discrimination, 47% experienced a high study pace, 19% experienced low social cohesion and 13% perceived the physical study environment as poor (Table 1). In the MSKHC, the mean age was 24.5

For peer review only

years and 59% were female. Eight percent had been exposed to discrimination, 55% experienced a high study pace, 24% experienced low social cohesion and 15% perceived the physical study environment as poor (Table 1). Characteristics of all study participants stratified by exposure status is presented in Table 2 and 3.

Table 1. Characteristics of the participants in the two cohorts

	**Mental health cohort (n = 2503)**	**Musculoskeletal health cohort (n = 2871)**
Age, mean (SD)	25.0 (6.4)	24.5 (6.1)
Gender, n (%)		
Female	1427 (57%)	1698 (59%)
Male	1065 (43%)	1157 (40%)
Other	11 (0%)	16 (1%)
Living Status, n (%)		
Alone	695 (28%)	806 (28%)
With others	1808 (72%)	2065 (72%)
Education type, n (%)		
Medical/Health	1184 (47%)	1271 (44%)
Technical	989 (40%)	1231 (43%)
Social sciences/humanities	228 (9%)	243 (9%)
Economic	59 (2%)	86 (3%)
Other	43 (2%)	40 (1%)
Highest parental education, n (%)		
University	1826 (73%)	2097 (73%)
Below university	677 (27%)	774 (27%)
Study year, n (%)		
First Year	1032 (41%)	1207 (42%)
Above First Year	1471 (59 %)	1664 (58%)
Place of birth, n (%)		
Sweden	1995 (80%)	2247 (78%)
Scandinavia	90 (4%)	95 (3%)
Europe	151 (6%)	172 (6%)
Outside Europe	267 (11%)	357 (12%)
Discrimination, n (%)	174 (7%)	239 (8%)
High study pace, n (%)	1180 (47%)	1587 (55%)
Low social cohesion, n (%)	479 (19%)	690 (24 %)
Poor physical study environment, n (%)	327 (13%)	437 (15%)

For peer review only

Table 2. Characteristics of the participants in the mental health cohort by exposure status.

	**Discrimination**		**High study pace**		**Low social cohesion [a]**		**Poor physical environment**	
	Unexposed (n=2329)	***Exposed (n=174)***	***Unexposed (n=1323)***	***Exposed (n=1180)***	***Unexposed (n=1986)***	***Exposed (n=479)***	***Unexposed (n=2176)***	***Exposed (n=327)***
Age, mean (SD)	24.9 (6.3)	25.9 (7.1)	25.2 (6.5)	24.8 (6.4)	24.7 (6.2)	25.4 (6.6)	24.8 (6.3)	26.5 (7.1)
Gender, n (%)								
Female	1301 (56%)	126 (72%)	680 (51%)	747 (63%)	1145 (58%)	254 (53%)	1231 (57%)	196 (60%)
Male	1019 (44%)	46 (26%)	639 (48%)	426 (36%)	834 (42%)	221 (46%)	936 (43%)	129 (39%)
Other [b]	-	-	-	-	-	-	-	-
Living Status, n (%)								
Alone	637 (27%)	58 (33%)	363 (27%)	332 (28%)	549 (28%)	140 (29%)	605 (28%)	90 (28%)
With others	1692 (73%)	116 (67%)	960 (73%)	848 (72%)	1437 (72%)	339 (71%)	1571 (72%)	237 (72%)
Education program, n (%)								
Medical/Health	1104 (47%)	80 (46%)	708 (54%)	476 (40%)	976 (49%)	189 (40%)	1019 (47%)	165 (51%)
Technical	926 (40%)	63 (36%)	451 (34%)	538 (46%)	774 (39%)	202 (42%)	891 (41%)	98 (30%)
Social sciences/ humanities	205 (9%)	23 (13%)	105 (8%)	123 (10%)	148 (8%)	74 (15%)	193 (9%)	35 (11%)
Economic [b]	-	-	-	-	-	-	-	-
Other[b]	-	-	-	-	-	-	-	-
Highest parental education, n (%)								
University	1706 (73%)	120 (69%)	967 (73%)	859 (73%)	1479 (74%)	324 (68%)	1592 (73%)	234 (72%)
Below university	623 (27%)	54 (31%)	356 (27%)	321 (27%)	507 (26%)	155 (32%)	584 (27%)	93 (28%)
Study year, n (%)								
First Year	984 (42%)	48 (28%)	507 (38%)	525 (44%)	840 (42%)	172 (36%)	919 (42%)	113 (35%)
Above First Year	1345 (58%)	126 (72%)	816 (62%)	655 (66%)	1146 (58%)	307 (64%)	1257 (58%)	214 (65%)
Place of birth, n (%)								
Sweden	1873 (80%)	122 (70%)	1050 (79%)	945 (80%)	1599 (81%)	371 (78%)	1721 (79%)	274 (84%)
Scandinavia [b]	-	-	52 (4%)	38 (3%)	77 (4%)	12 (3%)	-	-
Europe	-	-	83 (6%)	68 (6%)	108 (5%)	37 (8%)	-	-
Outside Europe	229 (10%)	38 (22%)	138 (10%)	129 (11%)	202 (10%)	59 (12%)	231 (11%)	36 (11%)

[a] 38 persons excluded due to studying at programs not organized in study groups
[b] Some numbers are not presented due to low cell counts

For peer review only

Table 3. Characteristics of the participants in the musculoskeletal health cohort by exposure status.

	**Discrimination**		**High study pace**		**Low social Cohesion**[a]		**Poor physical environment**	
	Unexposed (n=2632)	***Exposed (n=239)***	***Unexposed (n=1284)***	***Exposed (n=1587)***	***Unexposed (n=2133)***	***Exposed (n=690)***	***Unexposed (n=2434)***	***Exposed (n=437)***
Age, mean (SD)	24.5 (6.1)	24.7 (6.1)	24.8 (6.2)	24.3 (6.0)	24.4 (6.0)	24.6 (6.0)	24.3 (6.0)	25.5 (6.8)
Gender, n (%)								
Female	1536 (58%)	162 (67%)	667 (52%)	1031 (65%)	1283 (60%)	384 (55%)	1426 (59%)	272 (62%)
Male	1084 (41%)	73 (31%)	610 (48%)	547 (35%)	840 (40%)	300 (44%)	998 (41%)	159 (36%)
Other[b]	-	-	-	-	-	-	-	-
Living Status, n (%)								
Alone	732 (28%)	74 (31%)	346 (27%)	460 (29%)	598 (28%)	202 (29%)	687 (28%)	119 (27%)
With others	1900 (72%)	165 (69%)	938 (73%)	1127 (71%)	1535 (72%)	488 (71%)	1747 (72%)	318 (73%)
Education program, n (%)								
Medical/Health	1176 (45%)	95 (40%)	668 (52%)	603 (38%)	1001 (47%)	252 (37%)	1074 (44%)	197 (46%)
Technical	1130 (43%)	101 (42%)	461 (36%)	770 (49%)	869 (41%)	336 (49%)	1080 (44%)	151 (35%)
Social sciences/ humanities	214 (8%)	29 (12%)	98 (7%)	145 (9%)	156 (7%)	83 (12%)	196 (8%)	47 (11%)
Economic [b]	-	-	30 (2%)	55 (4%)	-	-	-	-
Other [b]	-	-	27 (2%)	13 (1%)	-	-	-	-
Highest parental education, n (%)								
University	1935 (74%)	162 (68%)	948 (74%)	1149 (72%)	1589 (75%)	477 (69%)	1781 (73%)	316 (72%)
Below university	697 (26%)	77 (32%)	336 (26%)	438 (28%)	544 (26%)	213 (31%)	653 (27%)	121 (28%)
Study year, n (%)								
First Year	1122 (43%)	85 (36%)	491 (38%)	716 (45%)	935 (44%)	248 (36%)	1044 (43%)	163 (37%)
Above First Year	1510 (57%)	154 (64%)	793 (62%)	871 (55%)	1198 (56%)	442 (64%)	1390 (57%)	274 (63%)
Place of birth, n (%)								
Sweden	2083 (79%)	162 (68%)	1015 (79%)	1232 (78%)	1695 (80%)	520 (76%)	1891 (78%)	356 (82%)
Scandinavia [b]	-	-	47 (4%)	48 (3%)	76 (4%)	18 (3%)	-	-
Europe [b]	-	-	80 (6%)	92 (6%)	117 (6%)	50 (7%)	-	-
Outside Europe	303 (12%)	54 (23%)	142 (11%)	215 (14%)	245 (12%)	102 (15%)	302 (12%)	55 (12%)

[a] 48 persons excluded due to studying at programs not organized in study groups

[b] Some numbers are not presented due to low cell count

For peer review only

Risk analysis

The number of outcome events at each time-period is presented in Supplemental eTable 6. When comparing the hazard of the outcomes between exposed and unexposed participants, discrimination gave adjusted HRs of 1.75 (95% CI: 1.40 – 2.19) for moderate or worse mental health problems and 1.39 (95% CI: 1.12 – 1.72) for activity-limiting musculoskeletal problems in any body location. High study pace gave adjusted HRs of 1.70 (95% CI: 1.48 – 1.94) for mental health problems, and 1.25 (95% CI: 1.09 – 1.43) for activity-limiting musculoskeletal problems. Low social cohesion gave adjusted HRs of 1.51 (95% CI: 1.29 – 1.77) for mental health problems and 1.08 (95% CI: 0.93 – 1.25) for activity-limiting musculoskeletal problems. Poor physical environment gave adjusted HRs of 1.20 (95% CI: 0.99 – 1.45) for mental health problems, and 1.20 (95% CI: 1.01 – 1.43) for activity-limiting musculoskeletal problems (Table 4).

Table 4. Hazard ratios of mental health problems and activity-limiting musculoskeletal problems in any body location comparing exposed to unexposed for different aspects of a poor study environment.

	**Mental health problems**		**Activity-limiting musculoskeletal problems**	
	Crude HR (95% CI)	*Adjusted HR [a] (95% CI)*	*Crude HR (95% CI)*	*Adjusted HR [a] (95% CI)*
Discrimination	1.89 (1.52 – 2.35)	1.75 (1.40 – 2.19)	1.43 (1.16 – 1.77)	1.39 (1.12 – 1.72)
High study pace	1.83 (1.61 – 2.09)	1.70 (1.48 – 1.94)	1.21 (1.06 – 1.38)	1.25 (1.09 – 1.43)
Low social cohesion	1.55 (1.33 – 1.80)	1.51 (1.29 – 1.77)	1.06 (0.92 – 1.23)	1.08 (0.93 – 1.25)
Poor physical environment	1.17 (0.97 – 1.41)	1.20 (0.99 – 1.45)	1.23 (1.04 – 1.46)	1.20 (1.01 – 1.43)

[a] Adjusted for gender, age, living situation, education type, year of study, place of birth and highest level of parental education.

Sensitivity analyses

Additionally *adjusting for baseline levels of depression, anxiety, and stress* symptoms gave the following HRs for mental health problems: Discrimination (HR: 1.34 [95% CI: 1.07 - 1.69]); High study

pace (HR: 1.18 [95% CI: 1.02 - 1.36]); Low social cohesion (HR: 1.28 [95% CI: 1.09 - 1.50]): Poor physical environment (HR: 1.02 [0.84 - 1.24]). For the outcome activity-limiting musculoskeletal problems, the HRs adjusted for baseline levels of depression, anxiety, and stress symptoms were: Discrimination (HR: 1.28 [95% CI: 1.03-1.60]); High study pace (HR: 1.13 [95% CI: 0.98-1.30]); Low social cohesion (HR: 1.01 [95% CI: 0.87-1.18]); Poor physical environment (HR: 1.15 [95% CI: 0.97-1.37]). The main results were not confounded by if the exposures were measured before or during *the COVID-19 pandemic* (Supplemental eTable 1, Table 4). *Gender stratified analyses* showed some potential differences in the strength of the associations between the exposures and the outcomes between men and women, although uncertain with overlapping confidence intervals (Supplemental eTable 2). Sensitivity analysis for *selection bias* showed that all exposures were weakly related to loss to follow-up (Supplemental eTable 3). The E-value analysis indicated that for some exposures, relatively weak associations between the outcome and missingness could affect the external validity of the results (Supplemental eTable 4). Treating each response category as a separate exposure level indicated *dose – response relationships* between high study pace and both mental health problems and activity-limiting musculoskeletal problems as well as between low social cohesion and mental health problems (Supplemental eTable 5).

Discussion

Several aspects of the study environment were associated with the incidence of mental health problems and activity-limiting musculoskeletal problems in this sample of Swedish university students, even after adjustment for multiple potential confounders. Discrimination at university was experienced by 7-8% of our sample and associated with the incidence of both mental health problems and activity-limiting musculoskeletal problems. Our results are in line with previous findings on discrimination and mental health (16, 17) but highlights that discrimination in the university setting may also be of importance in relation to activity-limiting musculoskeletal problems. The association was somewhat weaker for activity-limiting musculoskeletal problems than for mental

For peer review only

health problems, which is in line with earlier research suggesting that discrimination increases the risk of chronic pain by mediation from psychological distress (18).

High study pace was commonly experienced in our sample and associated with the incidence of both mental health problems and activity-limiting musculoskeletal problems, which corroborates earlier research (19, 20). Sensitivity analyses indicated a dose-response relationship between high study pace and both mental health problems and activity-limiting musculoskeletal problems. High study pace was, like discrimination, more strongly associated with the incidence of mental health problems than with activity-limiting musculoskeletal problems.

Low social cohesion at university was experienced by about a fifth of our sample and associated with higher incidence of mental health problems, with sensitivity analyses indicating a dose-response relationship. This association has not, to our knowledge, been studied among university students previously, but the results are in line with research showing associations between lack of school cohesion and mental health problems among adolescents (23). Low social cohesion was also associated with higher incidence of activity-limiting musculoskeletal problems, although the association was weak, and the confidence intervals did not exclude an effect in the opposite direction. Although inconclusive, these results are in line with earlier research from working populations (24).

The physical environment at university was perceived as good by the majority of our sample. However, perceiving the environment as poor was associated with a somewhat higher incidence of both mental health problems and activity-limiting musculoskeletal problems, although for mental health problems the confidence intervals did not exclude an association in the opposite direction. These findings support earlier findings of association between physical aspects of the study environment and pain (25, 26).

Gender stratified analyses showed that there may be differences between women and men in how strongly these exposures are related with the outcomes. For instance, exposure to high study pace

For peer review only

and low social cohesion were more strongly associated to mental health problems among men than among women. However, these gender differences are uncertain due to overlapping confidence intervals

Mental health problems and musculoskeletal problems are some of the most pressing public health concerns for young adults (1) and are highly prevalent among university students (2-4). Our results indicate that aspects of the study environment may be an important target for universities trying to tackle the high levels of mental health problems and activity-limiting musculoskeletal problems among university students. Addressing poor study environment issues might to some extent reduce incidence of mental health problems and activity-limiting musculoskeletal problems among university students. Future research on the effect of intervening on these study environment factors on mental health and musculoskeletal problems is warranted.

Strengths and limitations

This study has several strengths but also limitations. We were able to recruit a large sample of university students, which allowed us to study the associations between different study environment exposures and the outcomes with good precision. Further, the longitudinal design and the control for multiple potential confounders strengthens the level of evidence for a causal association.

There are, however, limitations hindering us from ascertaining causality. First, there is still the possibly unmeasured and residual confounding. Factors such as genetic vulnerability and neuroticism may have affected both the exposures and the outcome and thereby confounded our estimates. We assumed that subclinical symptoms at baseline were on the causal pathway from the exposure to the outcomes. If this assumption does not hold, we have the risk of reverse causality. Our sensitivity analysis showed that all associations weakened when controlling for baseline levels of depression, anxiety and stress symptoms, as expected both if subclinical symptoms were mediators or confounders. However, all exposures, except poor physical environment, were still associated with the incidence of mental health problems, suggesting that reverse causality is unlikely to explain away

For peer review only

the findings. For the incidence of activity-limiting musculoskeletal problems, the confidence intervals of all exposures except for discrimination, included the null when controlling for baseline mental health symptoms, leaving these associations more vulnerable to confounding by prior mental health problems. Second, since there was loss to follow-up in this study, our estimates may be affected by selection bias. Sensitivity analyses showed that all exposures were rather weakly associated to loss to follow-up, making it unlikely that selection bias would have affected the internal validity to any large extent (36). Selection bias could have affected the external validity (i.e. generalizability) of our results, and potentially explain away some of the weaker associations (Supplemental eTable 5), although it is hard to determine the direction of this potential bias. Third, the outcomes were measured using valid questionnaires, which limits the risk for misclassification in the outcomes. The exposures were measured using single item questions, and it is possible that misclassification of the exposures is present. We believe that this error is most likely to be non-differential, which would attenuate the true associations. However, since self-rated measures were used, the risk of differential misclassification cannot be ruled out, (e.g. due to common source bias), which could lead to overestimation of the associations. The use of single item exposures also limits our ability to differentiate between different aspects of the exposures. For instance, our measure of the perceived physical environment measure is quite crude and does not differentiate between different aspects of the physical environment (e.g. ergonomic workspaces, lighting, noise etc.).

Most of the data used in this study was collected after the COVID-19 pandemic broke out. During this period univeristy education in Sweden was mostly delivered online. The switch to online education clearly affected the study environment, but effects on student health are uncertain (38-40). Sensitivity analyses showed that our estimates were unaffected by adjustments for whether the exposures were measured before or during the pandemic. Still, it is possible that the mechanism linking study environment factors to mental and musculoskeletal health could have been different during the pandemic. For instance, physical study environment may have had larger effects on the outcomes, had the students been followed during a time-period when they spent more time on

For peer review only

campus. Thus, it is not certain to what extent these results, especially those regarding the physical study environment, will generalize to non-pandemic periods. Further, our sample was restricted to certain universities, and to participants agreeing to participate, and does not fully represent the overall Swedish student population. It is therefore unclear how well these results may generalize to other populations. We believe, however, that the associations observed in this study are similar to those in other populations at other time-periods, a belief supported by the alignment of our results to those of prior research conducted in different populations before the COVID-19 pandemic (16-20, 23-26).

Conclusions

Several aspects of the study environment were associated with the incidence of mental health problems and activity-limiting musculoskeletal problems in our sample of Swedish university students, after controlling for multiple potential confounders.

For peer review only

Figure 1. Flowchart of the inclusion of participants. MHC = Mental health cohort; MSKHC = Musculoskeletal health cohort.

Acknowledgements

We thank Pierre Côté, Klara Edlund and Tobias Sundberg for their important contributions in planning and performing the SUN-study data collection. We would also like to express our appreciation to all the participating students for their contribution to the SUN-study.

Funding

This research project was funded by the Swedish Research Council for Health, Working Life and Welfare (FORTE), grant number FORTE2018-00402. The SUN-study also received financial support from the Public Health Agency of Sweden.

For peer review only

Competing interests

The authors report no competing interests.

Contributorship statement

Conceptualization – Fred Johansson, Jessica Billquist, Hanna Andreasson, and Eva Skillgate

Data curation – Fred Johansson

Formal Analysis – Fred Johansson

Funding acquisition – Eva Skillgate

Investigation – Fred Johansson, Clara Onell, and Eva Skillgate

Methodology – Fred Johansson and Eva Skillgate

Project administration – Eva Skillgate

Supervision – Anne H Berman and Eva Skillgate

Writing – original draft – Fred Johansson, Jessica Billquist, Hanna Andreasson and Eva Skillgate

Writing – review & editing – Fred Johansson, Jessica Billquist, Hanna Andreasson, Irene Jensen, Clara Onell, Anne H Berman, and Eva Skillgate

Data Availability Statement

The dataset analyzed in the current study is not publicly available due to secondary confidentiality and privacy of the participants.

Ethics Approval

The study was approved by the Swedish Ethical Review Authority (2019-03276), and all participants provided informed consent electronically before entering the study.

References

1. Mokdad AH, Forouzanfar MH, Daoud F, Mokdad AA, El Bcheraoui C, Moradi-Lakeh M, et al. Global burden of diseases, injuries, and risk factors for young people's health during 1990-2013: a systematic analysis for the Global Burden of Disease Study 2013. Lancet. 2016;387(10036):2383-401.
2. Rotenstein LS, Ramos MA, Torre M, Segal JB, Peluso MJ, Guille C, et al. Prevalence of Depression, Depressive Symptoms, and Suicidal Ideation Among Medical Students: A Systematic Review and Meta-Analysis. JAMA: Journal of the American Medical Association. 2016;316(21):2214-36.
3. Ibrahim AK, Kelly SJ, Adams CE, Glazebrook C. A systematic review of studies of depression prevalence in university students. J Psychiatr Res. 2013;47(3):391-400.
4. Sheldon E, Simmonds-Buckley M, Bone C, Mascarenhas T, Chan N, Wincott M, et al. Prevalence and risk factors for mental health problems in university undergraduate students: A systematic review with meta-analysis. J Affect Disord. 2021;287:282-92.
5. Auerbach RP, Alonso J, Axinn WG, Cuijpers P, Ebert DD, Green JG, et al. Mental disorders among college students in the World Health Organization World Mental Health Surveys. Psychol Med. 2016;46(14):2955-70.
6. Keyes CL, Eisenberg D, Perry GS, Dube SR, Kroenke K, Dhingra SS. The relationship of level of positive mental health with current mental disorders in predicting suicidal behavior and academic impairment in college students. J Am Coll Health. 2012;60(2):126-33.
7. Rudman A, Gustavsson JP. Burnout during nursing education predicts lower occupational preparedness and future clinical performance: a longitudinal study. Int J Nurs Stud. 2012;49(8):988-1001.
8. Kim HJ, Boo S, Meeker TJ. Pain Prevalence, Management and Interference Among University Students in South Korea: An Exploratory Cross-Sectional Study. J Pain Res. 2021;14:2423-31.
9. Robertson D, Kumbhare D, Nolet P, Srbely J, Newton G. Associations between low back pain and depression and somatization in a Canadian emerging adult population. J Can Chiropr Assoc. 2017;61(2):96-105.

For peer review only

10. El Ansari W, Stock C, Snelgrove TUSHGS, Hu X, Parke S, Davies S, et al. Feeling Healthy? A Survey of Physical and Psychological Wellbeing of Students from Seven Universities in the UK. Int J Environ Res Public Health. 2011;8(5):1308-23.
11. Serbic D, Friedrich C, Murray R. Psychological, social and academic functioning in university students with chronic pain: A systematic review. J Am Coll Health. 2021:1-15.
12. Bair MJ, Robinson RL, Katon W, Kroenke K. Depression and Pain Comorbidity: A Literature Review. Arch Intern Med. 2003;163(20):2433-45.
13. Kroenke K, Wu J, Bair MJ, Krebs EE, Damush TM, Tu W. Reciprocal relationship between pain and depression: a 12-month longitudinal analysis in primary care. J Pain. 2011;12(9):964-73.
14. Bondesson E, Larrosa Pardo F, Stigmar K, Ringqvist Å, Petersson IF, Jöud A, et al. Comorbidity between pain and mental illness - Evidence of a bidirectional relationship. Eur J Pain. 2018;22(7):1304-11.
15. Gröjer A, Wikström Öbrand I, Elenäs J, Gillström P, Dryler H, Eriksson G. Studentspegeln 2016. 2017.
16. Pascoe EA, Smart Richman L. Perceived discrimination and health: a meta-analytic review. Psychol Bull. 2009;135(4):531-54.
17. Schmitt MT, Branscombe NR, Postmes T, Garcia A. The consequences of perceived discrimination for psychological well-being: a meta-analytic review. Psychol Bull. 2014;140(4):921-48.
18. Brown TT, Partanen J, Chuong L, Villaverde V, Chantal Griffin A, Mendelson A. Discrimination hurts: The effect of discrimination on the development of chronic pain. Soc Sci Med. 2018;204:1-8.
19. Barker ET, Howard AL, Villemaire-Krajden R, Galambos NL. The Rise and Fall of Depressive Symptoms and Academic Stress in Two Samples of University Students. J Youth Adolesc. 2018;47(6):1252-66.
20. Nerdrum P, Rustøen T, Helge Rønnestad M. Psychological Distress Among Nursing, Physiotherapy and Occupational Therapy Students: A Longitudinal and Predictive Study. Scandinavian Journal of Educational Research. 2009;53(4):363-78.
21. Grimby-Ekman A, Andersson EM, Hagberg M. Analyzing musculoskeletal neck pain, measured as present pain and periods of pain, with three different regression models: a cohort study. BMC Musculoskelet Disord. 2009;10:73.
22. Leigh-Hunt N, Bagguley D, Bash K, Turner V, Turnbull S, Valtorta N, et al. An overview of systematic reviews on the public health consequences of social isolation and loneliness. Public Health. 2017;152:157-71.
23. Santini ZI, Pisinger VSC, Nielsen L, Madsen KR, Nelausen MK, Koyanagi A, et al. Social Disconnectedness, Loneliness, and Mental Health Among Adolescents in Danish High Schools: A Nationwide Cross-Sectional Study. Front Behav Neurosci. 2021;15:632906.
24. Hoogendoorn WE, van Poppel MN, Bongers PM, Koes BW, Bouter LM. Systematic review of psychosocial factors at work and private life as risk factors for back pain. Spine (Phila Pa 1976). 2000;25(16):2114-25.
25. Ogunlana MO, Govender P, Oyewole OO. Prevalence and patterns of musculoskeletal pain among undergraduate students of occupational therapy and physiotherapy in a South African university. Hong Kong Physiother J. 2021;41(1):35-43.
26. Gheysvandi E, Dianat I, Heidarimoghadam R, Tapak L, Karimi-Shahanjarini A, Rezapur-Shahkolai F. Neck and shoulder pain among elementary school students: prevalence and its risk factors. BMC Public Health. 2019;19(1):1299.
27. Edlund K, Sundberg T, Johansson F, Onell C, Rudman A, Holm LW, et al. Sustainable UNiversity Life (SUN) study: protocol for a prospective cohort study of modifiable risk and prognostic factors for mental health problems and musculoskeletal pain among university students. BMJ Open. 2022;12(4):e056489.
28. Henry JD, Crawford JR. The short-form version of the Depression Anxiety Stress Scales (DASS-21): construct validity and normative data in a large non-clinical sample. Br J Clin Psychol. 2005;44(Pt 2):227-39.

For peer review only

29. Alfonsson S, Wallin E, Maathz P. Factor structure and validity of the Depression, Anxiety and Stress Scale-21 in Swedish translation. J Psychiatr Ment Health Nurs. 2017;24(2-3):154-62.
30. Kuorinka I, Jonsson B, Kilbom A, Vinterberg H, Biering-Sørensen F, Andersson G, et al. Standardised Nordic questionnaires for the analysis of musculoskeletal symptoms. Appl Ergon. 1987;18(3):233-7.
31. Hefner J, Eisenberg D. Social Support and Mental Health Among College Students. Am J Orthopsychiatry. 2009;79(4):491-9.
32. Bewick B, Koutsopoulou G, Miles J, Slaa E, Barkham M. Changes in undergraduate students' psychological well-being as they progress through university. Studies in Higher Education. 2010;35(6):633-45.
33. Beiter R, Nash R, McCrady M, Rhoades D, Linscomb M, Clarahan M, et al. The prevalence and correlates of depression, anxiety, and stress in a sample of college students. J Affect Disord. 2015;173:90-6.
34. Hernan MA, Robins JM. Causal Inference: What If. 1st ed. Boca Raton: Chapman & Hall/CRC; 2020.
35. Allison PD. Discrete-Time Methods for the Analysis of Event Histories. Sociol Methodol. 1982;13:61-98.
36. Lu H, Cole SR, Howe CJ, Westreich D. Toward a Clearer Definition of Selection Bias When Estimating Causal Effects. Epidemiology. 2022;33(5):699-706.
37. Smith LH, VanderWeele TJ. Bounding Bias Due to Selection. Epidemiology. 2019;30(4):509-16.
38. Johansson F, Côté P, Hogg-Johnson S, Skillgate E. Depression, anxiety and stress among Swedish university students during the second and third waves of COVID-19: A cohort study. Scandinavian Journal of Public Health. 2021:14034948211031402.
39. Johansson F, Cote P, Hogg-Johnson S, Rudman A, Holm LW, Grotle M, et al. Depression, anxiety and stress among Swedish university students before and during six months of the COVID-19 pandemic: A cohort study. Scand J Public Health. 2021;49(7):741-9.
40. Berman AH, Bendtsen M, Molander O, Lindfors P, Lindner P, Granlund L, et al. Compliance with recommendations limiting COVID-19 contagion among university students in Sweden: associations with self-reported symptoms, mental health and academic self-efficacy. Scand J Public Health. 2022;50(1):70-84.

For peer review only

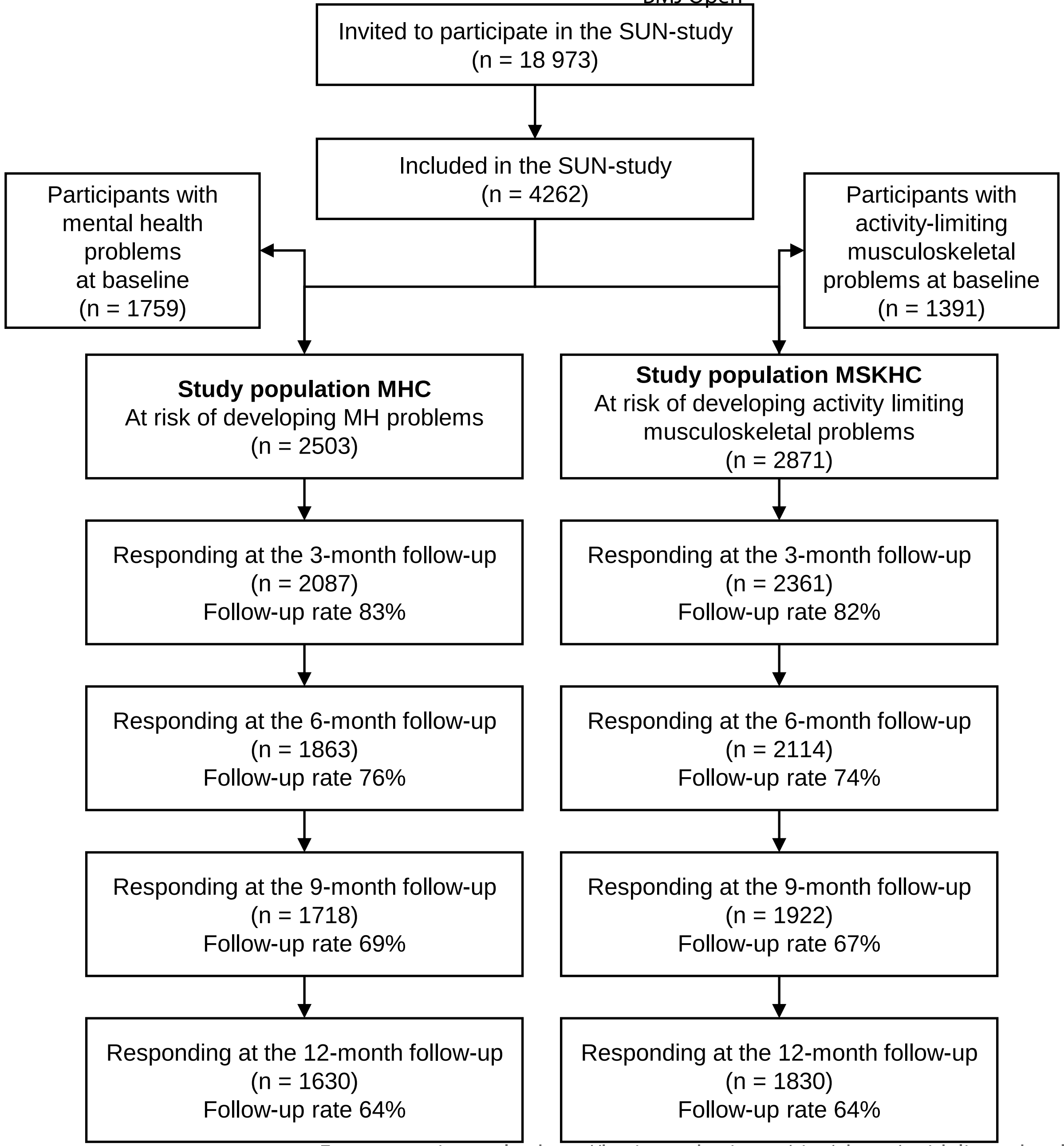

For peer review only - http://bmjopen.bmj.com/site/about/guidelines.xhtml

Supplemental material

Johansson, F., Billquist, J., Andreasson, H., Jensen, I., Onell, C., Berman, A. H., Skillgate, E. Study Environment and the Incidence of Mental Health Problems and Activity-Limiting Musculoskeletal Problems among University Students: The SUN Cohort Study

eTable 1. COVID-19 adjusted hazard ratios of mental health problems and activity-limiting musculoskeletal problems in any body location comparing exposed to unexposed

eTable 2. Gender specific hazard ratios of mental health problems and activity-limiting musculoskeletal problems in any body location comparing exposed to unexposed

eTable 3. Association between baseline exposure and dropout at the 12-month follow-up

eTable 4. E-values for selection bias giving the minimum association between the outcome and missingness among the unexposed needed to move the HR estimates to the null

eTable 5. Dose response models for high study pace and low social cohesion with each response category as separate exposure level

eTable 6. Number of events and participants at risk at each time-period.

For peer review only

eTable 1. COVID-19 adjusted [a] hazard ratios of mental health problems and activity-limiting musculoskeletal problems in any body location comparing exposed to unexposed

	**Mental health problems**	**Activity-limiting musculoskeletal problems**
	Adjusted HR [b] *(95% CI)*	*Adjusted HR [b]* *(95% CI)*
Discrimination	1.76 (1.41 – 2.21)	1.38 (1.12 – 1.72)
High study pace	1.70 (1.48 – 1.95)	1.25 (1.09 – 1.43)
Low social cohesion	1.51 (1.29 – 1.76)	1.08 (0.93 – 1.26)
Poor physical environment	1.20 (0.99 – 1.46)	1.20 (1.01 – 1.43)

[a] these analyses include a dummy coded covariate indicating whether the baseline survey, where the exposures were measured, were collected before or after March 13, 2020, that is considered the start of the COVID-19 pandemic in Sweden.

[b] Adjusted for gender, age, living situation, education type, year of study, place of birth and highest level of parental education and covid-19 pandemic at baseline.

For peer review only

eTable 2. Gender specific hazard ratios of mental health problems and activity-limiting musculoskeletal problems in any body location comparing exposed to unexposed

	**Mental health problems** *Adjusted HR [a]* *(95% CI)*		**Activity-limiting musculoskeletal problems** *Adjusted HR [a]* *(95% CI)*	
	Women (n = 1263 [b])	Men (n = 927 [c])	Women (n = 1491 [d])	Men (n = 1007 [e])
Discrimination	1.79 (1.39 – 2.31)	1.85 (1.16 – 2.95)	1.31 (1.01 - 1.69)	1.58 (1.08 – 2.33)
High study pace	1.50 (1.27 – 1.78)	2.11 (1.67 – 2.66)	1.29 (1.09 - 1.53)	1.15 (0.92 – 1.43)
Low social cohesion	1.40 (1.15 – 1.71)	1.81 (1.41 – 2.32)	1.14 (0.94 – 1.38)	1.01 (0.79 – 1.29)
Poor physical environment	1.12 (0.89 – 1.42)	1.31 (0.94 – 1.83)	1.18 (0.95 – 1.46)	1.28 (0.95 – 1.71)

[a] Adjusted for age, living situation, education type, year of study, place of birth and highest level of parental education.

[b]1427 women were included in the cohort at baseline, but 164 did not provide any follow-up assessments, giving an analytic sample size of 1263.

[c] 1065 men were included in the cohort at baseline, but 138 did not provide any follow-up assessments, giving an analytic sample size of 927.

[d] 1698 women were included in the cohort at baseline, but 206 did not provide any follow-up assessments, giving an analytic sample size of 1491

[e] 1057 men were included in the cohort at baseline, but 150 did not provide any follow-up assessments, giving an analytic sample size of 1007.

For peer review only

**eTable 3**. Association between baseline exposure and dropout at the 12-month follow-up		
	**Mental health cohort**	**Musculoskeletal health cohort**
	RR (95% CI)	*RR (95% CI)*
Discrimination	1.15 (0.95 - 1.39)	1.27 (1.10 – 1.48)
High study pace	0.98 (0.88 – 1.09)	1.03 (0.94 – 1.14)
Low social cohesion	1.05 (0.92 – 1.20)	1.07 (0.96 – 1.19)
Poor physical environment	1.10 (0.95 – 1.28)	1.18 (1.04 – 1.33)
RR: Risk ratio		

**eTable 4**. E-values for selection bias giving the minimum association between the outcome and missingness among the unexposed needed to move the HR estimates [a] to the null [b]		
	**Mental health cohort**	**Musculoskeletal health cohort**
	RR (95% CI)	*RR (95% CI)*
Discrimination	1.47	1.26
High study pace	1.44	1.16
Low social cohesion	1.33	1.05
Poor physical environment	1.13	1.13
[a] This refers to the adjusted estimates presented in Table 5<br>[b] Assuming that missingness is higher for persons with the outcome among both exposed and unexposed<br>RR: Risk ratio		

For peer review only

**eTable 5**. Dose response models for high study pace and low social cohesion with each response category as a separate exposure level				
	**Mental health cohort**		**Musculoskeletal health cohort**	
	No. exp [a]	*Adjusted HR (95% CI)* [b]	*No. exp* [a]	*Adjusted HR (95% CI)* [b]
**High study pace**				
I feel that I have energy and time to cope with the study pace	1079	Ref.	1044	Ref.
The study pace is high. I do my best, but I am afraid that it is not enough	967	1.64 (1.43 - 1.88)	1201	1.20 (1.05 - 1.37)
The study pace breaks me. We get more assignments than I have time for, and I feel that I cannot make it	41	3.28 (2.25 - 4.77)	116	1.50 (1.13 - 1.98)
**Low social cohesion**				
The cohesion is pretty good. We help each other with the studies and there is always someone in the class to talk to	1662	Ref.	1779	Ref.
I do not think that our cohesion is good. We work independently, and it feels like it is not worth the inconvenience to try to change it	366	1.49 (1.27 - 1.70)	506	1.07 (0.92 - 1.25)
We do not have any cohesion, and no one takes any responsibility over our groupwork or the fellowship	26	2.12 (1.30 - 3.45)	45	1.14 (0.72 - 1.80)

[a] Refers to the number of exposed participants in the analytic sample at baseline.
[b] Adjusted for age, living situation, education type, year of study, place of birth and highest level of parental education.

For peer review only

**eTable 6.** Number of events and participants at risk at each time-period						
	**Mental health cohort**			**Musculoskeletal health cohort**		
Time-period	*Events (n)*	*At risk (n)* [a]	*Hazard* [b]	*Events (n)*	*At risk (n)* [a]	*Hazard* [b]
0-3 months	502	2087	0.24	475	2361	0.20
3-6 months	180	1436	0.13	234	1711	0.13
6-9 months	133	1163	0.11	142	1357	0.10
9-12 months	94	997	0.09	121	1184	0.10

[a] Number are not equal to the analytic sample since not all participants were observed at all time-points.

[b] The hazard equals the probability of the event conditional on not having had the event earlier (Events / At risk).

For peer review only

STROBE Statement—Checklist of items that should be included in reports of ***cohort studies***

	Item No	Recommendation	Page No
**Title and abstract**	1	(*a*) Indicate the study's design with a commonly used term in the title or the abstract<br>(*b*) Provide in the abstract an informative and balanced summary of what was done and what was found	1-2
**Introduction**			
Background/rationale	2	Explain the scientific background and rationale for the investigation being reported	4-5
Objectives	3	State specific objectives, including any prespecified hypotheses	5
**Methods**			
Study design	4	Present key elements of study design early in the paper	6
Setting	5	Describe the setting, locations, and relevant dates, including periods of recruitment, exposure, follow-up, and data collection	6
Participants	6	(*a*) Give the eligibility criteria, and the sources and methods of selection of participants. Describe methods of follow-up<br>(*b*) For matched studies, give matching criteria and number of exposed and unexposed	6
Variables	7	Clearly define all outcomes, exposures, predictors, potential confounders, and effect modifiers. Give diagnostic criteria, if applicable	6-8
Data sources/ measurement	8*	For each variable of interest, give sources of data and details of methods of assessment (measurement). Describe comparability of assessment methods if there is more than one group	6-8
Bias	9	Describe any efforts to address potential sources of bias	9
Study size	10	Explain how the study size was arrived at	-
Quantitative variables	11	Explain how quantitative variables were handled in the analyses. If applicable, describe which groupings were chosen and why	9
Statistical methods	12	(*a*) Describe all statistical methods, including those used to control for confounding<br>(*b*) Describe any methods used to examine subgroups and interactions<br>(*c*) Explain how missing data were addressed<br>(*d*) If applicable, explain how loss to follow-up was addressed<br>(*e*) Describe any sensitivity analyses	9
**Results**			
Participants	13*	(a) Report numbers of individuals at each stage of study—eg numbers potentially eligible, examined for eligibility, confirmed eligible, included in the study, completing follow-up, and analysed<br>(b) Give reasons for non-participation at each stage<br>(c) Consider use of a flow diagram	10 and figure 1
Descriptive data	14*	(a) Give characteristics of study participants (eg demographic, clinical, social) and information on exposures and potential confounders<br>(b) Indicate number of participants with missing data for each variable of interest<br>(c) Summarise follow-up time (eg, average and total amount)	10, table 1-4
Outcome data	15*	Report numbers of outcome events or summary measures over time	Table4

For peer review only

Main results	16	(*a*) Give unadjusted estimates and, if applicable, confounder-adjusted estimates and their precision (eg, 95% confidence interval). Make clear which confounders were adjusted for and why they were included<br>(*b*) Report category boundaries when continuous variables were categorized<br>(*c*) If relevant, consider translating estimates of relative risk into absolute risk for a meaningful time period	Table 4 and 5
Other analyses	17	Report other analyses done—eg analyses of subgroups and interactions, and sensitivity analyses	11
**Discussion**			
Key results	18	Summarise key results with reference to study objectives	11
Limitations	19	Discuss limitations of the study, taking into account sources of potential bias or imprecision. Discuss both direction and magnitude of any potential bias	12-13
Interpretation	20	Give a cautious overall interpretation of results considering objectives, limitations, multiplicity of analyses, results from similar studies, and other relevant evidence	14
Generalisability	21	Discuss the generalisability (external validity) of the study results	14
**Other information**			
Funding	22	Give the source of funding and the role of the funders for the present study and, if applicable, for the original study on which the present article is based	19

*Give information separately for exposed and unexposed groups.

Note: An Explanation and Elaboration article discusses each checklist item and gives methodological background and published examples of transparent reporting. The STROBE checklist is best used in conjunction with this article (freely available on the Web sites of PLoS Medicine at http://www.plosmedicine.org/, Annals of Internal Medicine at http://www.annals.org/, and Epidemiology at http://www.epidem.com/). Information on the STROBE Initiative is available at http://www.strobe-statement.org.

For peer review only

BMJ Open

Study Environment and the Incidence of Mental Health Problems and Activity-Limiting Musculoskeletal Problems among University Students: The SUN Cohort Study

Journal:	*BMJ Open*
Manuscript ID	bmjopen-2023-072178.R2
Article Type:	Original research
Date Submitted by the Author:	18-Aug-2023
Complete List of Authors:	Johansson, Fred; Sophiahemmet University, Department of Health Promotion Science<br>Billquist, Jessica; Uppsala Universitet, Department of Psychology<br>Andreasson, Hanna; Uppsala Universitet, Department of Psychology<br>Jensen, Irene; Karolinska Institutet, Institute of Environmental Medicine<br>Onell, Clara; Sophiahemmet University, Department of Health Promotion Science<br>Berman, Anne H.; Uppsala Universitet, Department of Psychology; Karolinska Institutet, Department of Clinical Neuroscience<br>Skillgate, Eva; Sophiahemmet University, Department of Health Promotion Science; Karolinska Institutet, Institute of Environmental Medicine
**Primary Subject Heading**:	Epidemiology
Secondary Subject Heading:	Mental health
Keywords:	MENTAL HEALTH, Depression & mood disorders < PSYCHIATRY, Musculoskeletal disorders < ORTHOPAEDIC & TRAUMA SURGERY, EPIDEMIOLOGY

SCHOLARONE™ Manuscripts

I, the Submitting Author has the right to grant and does grant on behalf of all authors of the Work (as defined in the below author licence), an exclusive licence and/or a non-exclusive licence for contributions from authors who are: i) UK Crown employees; ii) where BMJ has agreed a CC-BY licence shall apply, and/or iii) in accordance with the terms applicable for US Federal Government officers or employees acting as part of their official duties; on a worldwide, perpetual, irrevocable, royalty-free basis to BMJ Publishing Group Ltd ("BMJ") its licensees and where the relevant Journal is co-owned by BMJ to the co-owners of the Journal, to publish the Work in this journal and any other BMJ products and to exploit all rights, as set out in our licence.

The Submitting Author accepts and understands that any supply made under these terms is made by BMJ to the Submitting Author unless you are acting as an employee on behalf of your employer or a postgraduate student of an affiliated institution which is paying any applicable article publishing charge ("APC") for Open Access articles. Where the Submitting Author wishes to make the Work available on an Open Access basis (and intends to pay the relevant APC), the terms of reuse of such Open Access shall be governed by a Creative Commons licence – details of these licences and which Creative Commons licence will apply to this Work are set out in our licence referred to above.

Other than as permitted in any relevant BMJ Author's Self Archiving Policies, I confirm this Work has not been accepted for publication elsewhere, is not being considered for publication elsewhere and does not duplicate material already published. I confirm all authors consent to publication of this Work and authorise the granting of this licence.

For peer review only

Study Environment and the Incidence of Mental Health Problems and Activity-Limiting Musculoskeletal Problems among University Students: The SUN Cohort Study

Authors

Fred Johansson [1*]

Jessica Billquist[2]

Hanna Andreasson[2]

Irene Jensen[3]

Clara Onell [1]

Anne H Berman[2,4]

Eva Skillgate [1,3,5]

[1]Department of Health Promotion Science, Sophiahemmet University, Stockholm, Sweden

[2]Department of Psychology, Uppsala University, Uppsala, Sweden

[3]Unit of Intervention and Implementation Research for Worker Health, Institute of Environmental Medicine, Karolinska Institutet, Stockholm, Sweden

[4]Department of Clinical Neuroscience, Karolinska Institutet, Stockholm, Sweden

[5]Naprapathögskolan – Scandinavian College of Naprapathic Manual Medicine, Stockholm, Sweden

* Corresponding author: Fred Johansson, Sophiahemmet Högskola, Box 5605, 114 86 Stockholm, Sweden. Email: fred.johansson@shh.se. Phone: +46739516158

Word count: 3835

Keywords: Students; Mental Health; Musculoskeletal Problems; Perceived Discrimination; Social Cohesion; Stress, Psychological

For peer review only

Abstract

Objective. To determine the association between different aspects of study environment and the incidence of mental health problems and activity-limiting musculoskeletal problems. **Design, setting and participants.** We recruited a cohort of 4262 Swedish university students of whom 2503 (59%) were without moderate or worse mental health problems and 2871 (67%) without activity-limiting musculoskeletal problems at baseline. The participants were followed at five time-points over one year using web surveys. **Exposures.** Self-rated discrimination, high study pace, low social cohesion and poor physical environment measured at baseline. **Outcomes:** Self-rated mental health problems, defined as scoring above cut-off on any of the subscales of the Depression, anxiety, and stress scale. Self-rated activity-limiting musculoskeletal problems in any body location, assessed by the Nordic Musculoskeletal Questionnaire. **Statistical analysis.** Discrete survival-time analysis was used to estimate the hazard rate ratio (HR) of each exposure-outcome combination while adjusting for gender, age, living situation, education type, year of studies, place of birth and parental education as potential confounders. **Results.** For discrimination, adjusted HRs were 1.75 (95% CI: 1.40 – 2.19) for mental health problems and 1.39 (95% CI: 1.12 – 1.72) for activity-limiting musculoskeletal problems. For high study pace, adjusted HRs were 1.70 (95% CI: 1.48 – 1.94) for mental health problems and 1.25 (95% CI: 1.09 – 1.43) for activity-limiting musculoskeletal problems. For low social cohesion, adjusted HRs were 1.51 (95% CI: 1.29 – 1.77) for mental health problems and 1.08 (95% CI: 0.93 – 1.25) for activity-limiting musculoskeletal problems. For perceived poor physical study environment, adjusted HRs were 1.20 (95% CI: 0.99 – 1.45) for mental health problems and 1.20 (95% CI: 1.01 – 1.43) for activity-limiting musculoskeletal problems. **Conclusions.** Several aspects of the study environment were associated with the incidence of mental health problems and activity-limiting musculoskeletal problems in this sample of Swedish university students.

For peer review only

Strengths and limitations of this study

- We recruited a large sample of students, which enables us to estimate associations between the exposures and the outcomes with good precision.
- We followed the participants over time, and were able to adjust for multiple potential confounders, which strengthens the evidence level for causal associations.
- There may still be residual and unmeasured confounding and selection bias that could affect the associations.
- Exposures were measured with single items questions, which could increase the risk of misclassification.
- The sample was measured mostly during the COVID-19 pandemic and is not representative of Swedish university students overall, which may affect generalizability.

For peer review only

Introduction

Mental health problems and musculoskeletal problems, such as neck and back pain, are leading causes of years lost due to disability in young people globally (1), and common among university students (2-5). Previous studies show that 35.7% of students from high-income countries meet criteria for at least one common mental disorder (5). Mental health problems are associated with increased risk of student attrition (5), academic impairment (6), reduced occupational preparedness and future work performance (7). Musculoskeletal pain is also common among university students (8-10) and associated with poorer academic functioning and lower quality of life (11).

Mental health problems and pain commonly co-occur (12). This comorbidity has been suggested to be characterized by bidirectional causation, with pain influencing mental health and vice versa (13, 14). Given the high comorbidity and bidirectional influences, it is likely that these conditions share many risk factors. Some risk factors are likely to affect students and non-students alike. Others, like the exposure to university study environments, are specific to students, and pose an important research target for understanding and preventing student health problems. Study environment is a multifaceted construct with several physical and psychosocial aspects. This study focuses on four specific aspects: discrimination, study pace, social cohesion, and physical study environment.

Seven percent of Swedish students report experiencing discrimination during their studies (15). Perceived discrimination is associated with mental and physical ill health (16) and is negatively associated with subsequent psychological well-being even after controlling for baseline levels of psychological well-being (17). Brown et al (18) showed that the association between discrimination and chronic pain was mediated through psychological distress in a general population sample. However, little is known as to how discrimination occurring specifically at the university affects students' health.

Perceived stress and academic demands have been associated with higher levels of depressive symptoms and psychological distress among students in longitudinal studies (19, 20), and perceived

For peer review only

work/study demands with neck and upper back pain among young adults (21). However, few studies have focused specifically on high study pace and its associations to mental health and activity-limiting musculoskeletal problems.

Social isolation and loneliness are well-established risk factors for mental health problems in general (22), but less is known about the impact of low social cohesion within the study environment. For adolescents, a lack of social cohesion at school and lack of classmate support are cross-sectionally associated with lower psychological well-being and higher symptom levels of depression and anxiety (23). Low workplace social support has also been identified as a risk factor for back pain among working adults (24), but to our knowledge there is no studies on the association between social cohesion and activity-limiting musculoskeletal problems among university students.

Physical aspects of the study environment such as repetitive movement, prolonged sitting and sitting position are associated with musculoskeletal pain among university students (25) and school children (26). However, there is a lack of research into how students' perceptions of physical study environment is associated with subsequent mental health problems and activity-limiting musculoskeletal problems.

In this study, we aimed to determine the associations between four aspects of study environment: discrimination; high study pace; low social cohesion; and perceived poor physical environment, and the incidence of mental health problems and activity-limiting musculoskeletal problems among Swedish university students.

Method

Design and participants

This study is based on the Sustainable UNiversity life (SUN) cohort (27), a longitudinal study aiming to identify risk and prognostic factors for mental health problems and musculoskeletal pain and problems in university students. Participants were recruited between August 2019 and December

For peer review only

2021, from eight universities in the greater Stockholm area and Örebro. The targeted universities constitute a convenience sample, selected to cover a variety of educational programs mainly within medicine, business, technology, and social sciences. Full-time undergraduate or graduate students (up to and including masters' level) at selected educational programs with at least one full year of academic studies left before graduation were eligible for inclusion.

Eligible students were informed about the study during an in-class presentation and/or by email with a link to the baseline survey. Students agreeing to participate were followed every three months for one year using web surveys. For the aim of studying incidence of mental health problems and activity-limiting musculoskeletal problems in this study, two cohorts were established: 1) the mental health cohort (MHC) including participants without moderate or more severe mental health problems at baseline; and 2) the musculoskeletal health cohort (MSKHC) including participants without activity-limiting musculoskeletal problems at any body location at baseline (Figure 1).

The study was approved by the Swedish Ethical Review Authority (2019-03276), and all participants provided informed consent electronically before entering the study. More information about the study methodology and data collection is available in the study protocol (27).

Patient and public involvement

We involved students by conducting focus group interviews on health challenges of university students during the planning phase of the study. We have also had an ongoing dialogue with student health services, educational offices and student unions during the planning and conduct of the study, as well as for dissemination of the results. Students and university officials have been involved in the selection of exposures and outcomes, in recruitment and data collection phase, and some have participated in seminars on the results, organized by the research group.

For peer review only

Measures

Outcomes

Mental health problems were measured with the short-form Depression Anxiety Stress Scale (DASS-21) (28), consisting of 21 items including subscales on depression, anxiety, and stress. Each subscale comprises seven items rated on a scale from 0 (Did not apply to me at all) to 3 (Applied to me very much, or most of the time), with a recall time of one week. The items were summed to give subscale-scores ranging from 0 to 21. Participants scoring above the cut-off for moderate symptoms levels on any of the scales ($\geq$ 7 on the depression subscale, $\geq$ 6 on the anxiety subscale, $\geq$ 10 on the stress subscale) were considered to have mental health problems. DASS-21 has shown good psychometric properties among Swedish university students (29).

Activity-limiting musculoskeletal problems was measured with the Nordic Musculoskeletal Questionnaire (NMQ) (30). Participants were asked if they *"at any time during the last three months have been prevented from doing your normal activities (e.g. work, studies, household activities, hobbies) due to trouble with:"* ankles/feet, knee, hip, lower back, upper back, shoulder, elbow, hand, and/or neck. The question was responded to dichotomously (Yes/No) for each body location. Participants responding Yes for any of the body locations were considered to have activity-limiting musculoskeletal problems.

Exposures

All exposures were measured at baseline.

Discrimination was measured by the question: *"Have you ever during the past year felt discriminated by other students, teachers or administrative personnel due to:"* over nine categories: gender, gender identity, gender expression, ethnicity, religion or beliefs, sexual orientation, disabilities, age, other [15]. Participants answering "Yes" on at least one of the categories were asked to rate how severely the discrimination had affected them on a scale from 0 (Not at all) to 10 (Worst imaginable way). Participants with severity ratings from the 50th percentile (>3) were considered exposed. This cut-off was decided a-priori to identify more severe forms of discrimination.

For peer review only

Study pace was measured by the questions: *"Do you feel that you are coping with the study pace?"* with three response categories: (1) *"I feel that I have energy and time to cope with the study pace",* (2) "*The study pace is high. I do my best, but I am afraid that it is not enough"* and (3) "*The study pace breaks me. We get more assignments than I have time for, and I feel that I cannot make it"*. Participant's choosing categories 2 or 3 were considered exposed to high study pace.

Social cohesion was measured by the question: *"How good is the cohesion with your course mates?"*, with four response alternatives: (1) *"The cohesion is pretty good. We help each other with the studies and there is always someone in the class to talk to*", (2) *"I do not think that our cohesion is good. We work independently, and it feels like it is not worth the inconvenience to try to change it*", (3) *"We do not have any cohesion, and no one takes any responsibility over our groupwork or the fellowshi*p" and (4) *"My education is not organized around study groups or classes"*. Participants choosing categories 2 or 3 were considered exposed to low social cohesion. Participants choosing the fourth category were excluded from the risk analyses.

Perceived physical environment was measured by asking: "*How do you in general experience the physical study environment at your university? (For example, public areas, lunchroom, lecture halls, toilets, outside environment around campus.)"* and rated on a scale from 0 (Very bad) to 10 (Very good). Participants with ratings of 0-5 were considered exposed to a poor physical study environment.

Covariates

Potential confounders were selected based on previous research (e.g. (4, 31-33)) and directed acyclic graphs (34). We included the covariates gender (female, male, other), age (continuous), living situation (alone, with others), education type (medical/health, technical, social sciences/humanities, economic, other), year of studies (first, second, above), place of birth (Sweden, Scandinavia, Europe, outside Europe) and highest level of parental education (university, below university) as a proxy for socioeconomic status.

For peer review only

Statistical analysis

We used discrete-time survival analysis to estimate associations between baseline exposures and the hazard of each of the outcomes. A participant was considered at risk for the outcome until the outcome was first registered or until censoring due to loss to follow-up or end of follow-up, whichever came first.

We used generalized linear models with binomial error distributions and complementary log-log links to estimate the hazard ratio (HR) with 95% confidence intervals (CI) of the outcomes comparing the exposed to the unexposed. Separate models were built for each exposure – outcome combination. This modeling approach is equivalent to Cox proportional hazard models when assuming that the underlying time variable is continuous (35). Time was measured as four time-periods (0 to 3 months, 3 to 6 months, 6 to 9 months, and 9 to 12 months). Plotting the log hazard over the four time-periods showed a fairly linear relationship for both outcomes, and therefore the time-period variable was treated as continuous. The proportional hazard assumption was evaluated by including interaction terms between time-period and the exposures and performing likelihood ratio tests comparing models with and without interaction terms. These indicated better fits for models assuming proportional hazards for all exposure – outcome combinations, except for the model estimating the association between discrimination and mental health problems.

Sensitivity analyses

Several additional sensitivity analyses were run as detailed below. First, subclinical symptom levels of mental health problems at baseline were assumed to be on the causal pathway and thus not included as covariates in the main analyses. To assess the sensitivity of our analyses to this assumption, we performed sensitivity analyses where the continuous scores of the three DASS-21 subscales measured at baseline were included as covariates. Second, data collection took place before and during the COVID-19 pandemic, which may have affected both our exposures and

For peer review only

outcomes thus potentially leading to confounding. About half of the participants entered the SUN-study before the COVID-19 pandemic started in Sweden on March 13, 2020 (45% in MHC and 41% in MSKHC), and almost all outcome data was collected after the onset of the pandemic (92% in the MHC and 83% in the MSKHC). To assess the robustness of our results to potential confounding by the COVID-19 pandemic we reran the main analyses while including a covariate indicating if the baseline survey, where exposures were measured, was collected before or after March 13, 2020. Third, to assess potential effect modification by gender, gender stratified risk analyses were performed. Fourth, to assess potential selection bias we computed risk ratios (RR) of loss to follow-up at the 12-month follow-up between exposed and unexposed (36), and computed E-values defining the minimum association between missingness and the outcome, on the RR scale, needed to shift the point estimates of the main analysis to the null (37). Fifth, to investigate potential dose-response relationships for the exposures high study pace and low social cohesion we performed sensitivity analyses treating each response category as a separate exposure level.

All analyses were performed using R version 4.1.2.

Results

We included 4262 participants, of whom 2503 (59%) were without moderate or worse mental health problems at baseline and were included in the MHC and 2871 (67%) were without activity-limiting musculoskeletal problems in any body location at baseline and included in the MSKHC. A flow-chart of inclusion of study participants and follow-up rates is presented in Figure 1. Only participants responding to at least one follow-up could be included in the analytic sample for the main analysis, giving a total of 2200 participants in the MHC, and 2513 in the MSKHC.

In the MHC, the mean age was 25 years and 57% were females. Seven percent had been exposed to discrimination, 47% experienced a high study pace, 19% experienced low social cohesion and 13% perceived the physical study environment as poor (Table 1). In the MSKHC, the mean age was 24.5 years and 59% were female. Eight percent had been exposed to discrimination, 55% experienced a

For peer review only

high study pace, 24% experienced low social cohesion and 15% perceived the physical study environment as poor (Table 1). Characteristics of all study participants stratified by exposure status is presented in Table 2 and 3.

Table 1. Characteristics of the participants in the two cohorts

	Mental health cohort (n = 2503)	Musculoskeletal health cohort (n = 2871)
Age, mean (SD)	25.0 (6.4)	24.5 (6.1)
Gender, n (%)		
Female	1427 (57%)	1698 (59%)
Male	1065 (43%)	1157 (40%)
Other	11 (0%)	16 (1%)
Living Status, n (%)		
Alone	695 (28%)	806 (28%)
With others	1808 (72%)	2065 (72%)
Education type, n (%)		
Medical/Health	1184 (47%)	1271 (44%)
Technical	989 (40%)	1231 (43%)
Social sciences/humanities	228 (9%)	243 (9%)
Economic	59 (2%)	86 (3%)
Other	43 (2%)	40 (1%)
Highest parental education, n (%)		
University	1826 (73%)	2097 (73%)
Below university	677 (27%)	774 (27%)
Study year, n (%)		
First Year	1032 (41%)	1207 (42%)
Above First Year	1471 (59 %)	1664 (58%)
Place of birth, n (%)		
Sweden	1995 (80%)	2247 (78%)
Scandinavia	90 (4%)	95 (3%)
Europe	151 (6%)	172 (6%)
Outside Europe	267 (11%)	357 (12%)
Discrimination, n (%)	174 (7%)	239 (8%)
High study pace, n (%)	1180 (47%)	1587 (55%)
Low social cohesion, n (%)	479 (19%)	690 (24 %)
Poor physical study environment, n (%)	327 (13%)	437 (15%)

For peer review only

Table 2. Characteristics of the participants in the mental health cohort by exposure status.

	**Discrimination**		**High study pace**		**Low social cohesion [a]**		**Poor physical environment**	
	Unexposed (n=2329)	***Exposed (n=174)***	***Unexposed (n=1323)***	***Exposed (n=1180)***	***Unexposed (n=1986)***	***Exposed (n=479)***	***Unexposed (n=2176)***	***Exposed (n=327)***
Age, mean (SD)	24.9 (6.3)	25.9 (7.1)	25.2 (6.5)	24.8 (6.4)	24.7 (6.2)	25.4 (6.6)	24.8 (6.3)	26.5 (7.1)
Gender, n (%)								
Female	1301 (56%)	126 (72%)	680 (51%)	747 (63%)	1145 (58%)	254 (53%)	1231 (57%)	196 (60%)
Male	1019 (44%)	46 (26%)	639 (48%)	426 (36%)	834 (42%)	221 (46%)	936 (43%)	129 (39%)
Other [b]	-	-	-	-	-	-	-	-
Living Status, n (%)								
Alone	637 (27%)	58 (33%)	363 (27%)	332 (28%)	549 (28%)	140 (29%)	605 (28%)	90 (28%)
With others	1692 (73%)	116 (67%)	960 (73%)	848 (72%)	1437 (72%)	339 (71%)	1571 (72%)	237 (72%)
Education program, n (%)								
Medical/Health	1104 (47%)	80 (46%)	708 (54%)	476 (40%)	976 (49%)	189 (40%)	1019 (47%)	165 (51%)
Technical	926 (40%)	63 (36%)	451 (34%)	538 (46%)	774 (39%)	202 (42%)	891 (41%)	98 (30%)
Social sciences/ humanities	205 (9%)	23 (13%)	105 (8%)	123 (10%)	148 (8%)	74 (15%)	193 (9%)	35 (11%)
Economic [b]	-	-	-	-	-	-	-	-
Other[b]	-	-	-	-	-	-	-	-
Highest parental education, n (%)								
University	1706 (73%)	120 (69%)	967 (73%)	859 (73%)	1479 (74%)	324 (68%)	1592 (73%)	234 (72%)
Below university	623 (27%)	54 (31%)	356 (27%)	321 (27%)	507 (26%)	155 (32%)	584 (27%)	93 (28%)
Study year, n (%)								
First Year	984 (42%)	48 (28%)	507 (38%)	525 (44%)	840 (42%)	172 (36%)	919 (42%)	113 (35%)
Above First Year	1345 (58%)	126 (72%)	816 (62%)	655 (66%)	1146 (58%)	307 (64%)	1257 (58%)	214 (65%)
Place of birth, n (%)								
Sweden	1873 (80%)	122 (70%)	1050 (79%)	945 (80%)	1599 (81%)	371 (78%)	1721 (79%)	274 (84%)
Scandinavia [b]	-	-	52 (4%)	38 (3%)	77 (4%)	12 (3%)	-	-
Europe	-	-	83 (6%)	68 (6%)	108 (5%)	37 (8%)	-	-
Outside Europe	229 (10%)	38 (22%)	138 (10%)	129 (11%)	202 (10%)	59 (12%)	231 (11%)	36 (11%)

[a] 38 persons excluded due to studying at programs not organized in study groups
[b] Some numbers are not presented due to low cell counts

For peer review only

Table 3. Characteristics of the participants in the musculoskeletal health cohort by exposure status.

	**Discrimination**		**High study pace**		**Low social Cohesion**[a]		**Poor physical environment**	
	Unexposed (n=2632)	***Exposed (n=239)***	***Unexposed (n=1284)***	***Exposed (n=1587)***	***Unexposed (n=2133)***	***Exposed (n=690)***	***Unexposed (n=2434)***	***Exposed (n=437)***
Age, mean (SD)	24.5 (6.1)	24.7 (6.1)	24.8 (6.2)	24.3 (6.0)	24.4 (6.0)	24.6 (6.0)	24.3 (6.0)	25.5 (6.8)
Gender, n (%)								
Female	1536 (58%)	162 (67%)	667 (52%)	1031 (65%)	1283 (60%)	384 (55%)	1426 (59%)	272 (62%)
Male	1084 (41%)	73 (31%)	610 (48%)	547 (35%)	840 (40%)	300 (44%)	998 (41%)	159 (36%)
Other[b]	-	-	-	-	-	-	-	-
Living Status, n (%)								
Alone	732 (28%)	74 (31%)	346 (27%)	460 (29%)	598 (28%)	202 (29%)	687 (28%)	119 (27%)
With others	1900 (72%)	165 (69%)	938 (73%)	1127 (71%)	1535 (72%)	488 (71%)	1747 (72%)	318 (73%)
Education program, n (%)								
Medical/Health	1176 (45%)	95 (40%)	668 (52%)	603 (38%)	1001 (47%)	252 (37%)	1074 (44%)	197 (46%)
Technical	1130 (43%)	101 (42%)	461 (36%)	770 (49%)	869 (41%)	336 (49%)	1080 (44%)	151 (35%)
Social sciences/ humanities	214 (8%)	29 (12%)	98 (7%)	145 (9%)	156 (7%)	83 (12%)	196 (8%)	47 (11%)
Economic[b]	-	-	30 (2%)	55 (4%)	-	-	-	-
Other[b]	-	-	27 (2%)	13 (1%)	-	-	-	-
Highest parental education, n (%)								
University	1935 (74%)	162 (68%)	948 (74%)	1149 (72%)	1589 (75%)	477 (69%)	1781 (73%)	316 (72%)
Below university	697 (26%)	77 (32%)	336 (26%)	438 (28%)	544 (26%)	213 (31%)	653 (27%)	121 (28%)
Study year, n (%)								
First Year	1122 (43%)	85 (36%)	491 (38%)	716 (45%)	935 (44%)	248 (36%)	1044 (43%)	163 (37%)
Above First Year	1510 (57%)	154 (64%)	793 (62%)	871 (55%)	1198 (56%)	442 (64%)	1390 (57%)	274 (63%)
Place of birth, n (%)								
Sweden	2083 (79%)	162 (68%)	1015 (79%)	1232 (78%)	1695 (80%)	520 (76%)	1891 (78%)	356 (82%)
Scandinavia[b]	-	-	47 (4%)	48 (3%)	76 (4%)	18 (3%)	-	-
Europe[b]	-	-	80 (6%)	92 (6%)	117 (6%)	50 (7%)	-	-
Outside Europe	303 (12%)	54 (23%)	142 (11%)	215 (14%)	245 (12%)	102 (15%)	302 (12%)	55 (12%)

[a] 48 persons excluded due to studying at programs not organized in study groups
[b] Some numbers are not presented due to low cell count

For peer review only

Risk analysis

The number of outcome events at each time-period is presented in Supplemental eTable 1. When comparing the hazard of the outcomes between exposed and unexposed participants, discrimination gave adjusted HRs of 1.75 (95% CI: 1.40 – 2.19) for moderate or worse mental health problems and 1.39 (95% CI: 1.12 – 1.72) for activity-limiting musculoskeletal problems in any body location. High study pace gave adjusted HRs of 1.70 (95% CI: 1.48 – 1.94) for mental health problems, and 1.25 (95% CI: 1.09 – 1.43) for activity-limiting musculoskeletal problems. Low social cohesion gave adjusted HRs of 1.51 (95% CI: 1.29 – 1.77) for mental health problems and 1.08 (95% CI: 0.93 – 1.25) for activity-limiting musculoskeletal problems. Poor physical environment gave adjusted HRs of 1.20 (95% CI: 0.99 – 1.45) for mental health problems, and 1.20 (95% CI: 1.01 – 1.43) for activity-limiting musculoskeletal problems (Table 4).

Table 4. Hazard ratios of mental health problems and activity-limiting musculoskeletal problems in any body location comparing exposed to unexposed for different aspects of a poor study environment.

	**Mental health problems**		**Activity-limiting musculoskeletal problems**	
	Crude HR (95% CI)	*Adjusted HR [a] (95% CI)*	*Crude HR (95% CI)*	*Adjusted HR [a] (95% CI)*
Discrimination	1.89 (1.52 – 2.35)	1.75 (1.40 – 2.19)	1.43 (1.16 – 1.77)	1.39 (1.12 – 1.72)
High study pace	1.83 (1.61 – 2.09)	1.70 (1.48 – 1.94)	1.21 (1.06 – 1.38)	1.25 (1.09 – 1.43)
Low social cohesion	1.55 (1.33 – 1.80)	1.51 (1.29 – 1.77)	1.06 (0.92 – 1.23)	1.08 (0.93 – 1.25)
Poor physical environment	1.17 (0.97 – 1.41)	1.20 (0.99 – 1.45)	1.23 (1.04 – 1.46)	1.20 (1.01 – 1.43)

[a] Adjusted for gender, age, living situation, education type, year of study, place of birth and highest level of parental education.

Sensitivity analyses

Additionally *adjusting for baseline levels of depression, anxiety, and stress* symptoms gave the following HRs for mental health problems: Discrimination (HR: 1.34 [95% CI: 1.07 - 1.69]); High study

For peer review only

pace (HR: 1.18 [95% CI: 1.02 - 1.36]); Low social cohesion (HR: 1.28 [95% CI: 1.09 - 1.50]): Poor physical environment (HR: 1.02 [0.84 - 1.24]). For the outcome activity-limiting musculoskeletal problems, the HRs adjusted for baseline levels of depression, anxiety, and stress symptoms were: Discrimination (HR: 1.28 [95% CI: 1.03-1.60]); High study pace (HR: 1.13 [95% CI: 0.98-1.30]); Low social cohesion (HR: 1.01 [95% CI: 0.87-1.18]); Poor physical environment (HR: 1.15 [95% CI: 0.97-1.37]). Adding a variable indicating if exposures were measured before or during the COVID-19 pandemic did not affect the main results (Supplemental eTable 2, Table 4). *Gender stratified analyses* showed some potential differences in the strength of the associations between men and women for mental health, although differences are uncertain due to overlapping CI's. The point estimates for low social cohesion and high study pace were higher among men (Supplemental eTable 3). Sensitivity analysis for *selection bias* showed that all exposures were weakly related to loss to follow-up (Supplemental eTable 4). The E-value analysis indicated that for some exposures, relatively weak associations between the outcome and missingness could affect the external validity of the results (Supplemental eTable 5). Treating each response category as a separate exposure level indicated *dose – response relationships* between high study pace and both mental health problems and activity-limiting musculoskeletal problems as well as between low social cohesion and mental health problems (Supplemental eTable 6).

Discussion

Several aspects of the study environment were associated with the incidence of mental health problems and activity-limiting musculoskeletal problems in this sample of Swedish university students, even after adjustment for multiple potential confounders. Discrimination at university was experienced by 7-8% of our sample and associated with the incidence of both mental health problems and activity-limiting musculoskeletal problems. Our results are in line with previous findings on discrimination and mental health (16, 17) but highlights that discrimination in the university setting may also be of importance in relation to activity-limiting musculoskeletal problems. The association was somewhat weaker for activity-limiting musculoskeletal problems than for mental

For peer review only

health problems, which is in line with earlier research suggesting that discrimination increases the risk of chronic pain by mediation from psychological distress (18).

High study pace was commonly experienced in our sample and associated with the incidence of both mental health problems and activity-limiting musculoskeletal problems, which corroborates earlier research (19, 20). Sensitivity analyses indicated a dose-response relationship between high study pace and both mental health problems and activity-limiting musculoskeletal problems. High study pace was, like discrimination, more strongly associated with the incidence of mental health problems than with activity-limiting musculoskeletal problems.

Low social cohesion at university was experienced by about a fifth of our sample and associated with higher incidence of mental health problems, with sensitivity analyses indicating a dose-response relationship. This association has not, to our knowledge, been studied among university students previously, but the results are in line with research showing associations between lack of school cohesion and mental health problems among adolescents (23). Low social cohesion was also associated with higher incidence of activity-limiting musculoskeletal problems, although the association was weak, and the CI's did not exclude an effect in the opposite direction. Although inconclusive, these results are in line with earlier research from working populations (24).

The physical environment at university was perceived as good by the majority of our sample. However, perceiving the environment as poor was associated with a somewhat higher incidence of both mental health problems and activity-limiting musculoskeletal problems, although for mental health problems the CI's s did not exclude an association in the opposite direction. These findings support earlier findings of association between physical aspects of the study environment and pain (25, 26).

Gender stratified analyses showed that exposure to high study pace and low social cohesion may be more strongly associated to mental health problems among men than among women. However,

For peer review only

these gender differences are uncertain due to overlapping CI's and larger studies are needed to draw firm conclusions on these potential gender differences.

Mental health problems and musculoskeletal problems are some of the most pressing public health concerns for young adults (1) and are highly prevalent among university students (2-4). Our results indicate that aspects of the study environment may be an important target for universities trying to tackle the high levels of mental health problems and activity-limiting musculoskeletal problems among university students. Addressing poor study environment issues might to some extent reduce incidence of mental health problems and activity-limiting musculoskeletal problems among university students. Future research on the effect of intervening on these study environment factors on mental health and musculoskeletal problems is warranted.

Strengths and limitations

This study has several strengths but also limitations. We were able to recruit a large sample of university students, which allowed us to study the associations between different study environment exposures and the outcomes with good precision. Further, the longitudinal design and the control for multiple potential confounders strengthens the level of evidence for a causal association.

There are, however, limitations hindering us from ascertaining causality. First, there is still the possibly unmeasured and residual confounding. Factors such as genetic vulnerability and neuroticism may have affected both the exposures and the outcome and thereby confounded our estimates. We assumed that subclinical symptoms at baseline were on the causal pathway from the exposure to the outcomes. If this assumption does not hold, we have the risk of reverse causality. Our sensitivity analysis showed that all associations weakened when controlling for baseline levels of depression, anxiety and stress symptoms, as expected both if subclinical symptoms were mediators or confounders. However, all exposures, except poor physical environment, were still associated with the incidence of mental health problems, suggesting that reverse causality is unlikely to explain away the findings. For the incidence of activity-limiting musculoskeletal problems, the CI's of all exposures,

For peer review only

except for discrimination, included the null when controlling for baseline mental health symptoms, leaving these associations more vulnerable to confounding by prior mental health problems. Second, since there was loss to follow-up in this study, our estimates may be affected by selection bias. Sensitivity analyses showed that all exposures were rather weakly associated to loss to follow-up, making it unlikely that selection bias would have affected the internal validity to any large extent (36). Selection bias could have affected the external validity (i.e. generalizability) of our results, and potentially explain away some of the weaker associations (Supplemental eTable 6), although it is hard to determine the direction of this potential bias. Third, the outcomes were measured using valid questionnaires, which limits the risk for misclassification in the outcomes. The exposures were measured using single item questions, and it is possible that misclassification of the exposures is present. We believe that this error is most likely to be non-differential, which would attenuate the true associations. However, since self-rated measures were used, the risk of differential misclassification cannot be ruled out, (e.g. due to common source bias), which could lead to overestimation of the associations. The use of single item exposures also limits our ability to differentiate between different aspects of the exposures. For instance, our measure of the perceived physical environment measure is quite crude and does not differentiate between different aspects of the physical environment (e.g. ergonomic workspaces, lighting, noise etc.).

Most of the data used in this study was collected after the COVID-19 pandemic broke out. During this period univeristy education in Sweden was mostly delivered online. The switch to online education clearly affected the study environment, but effects on student health are uncertain (38-40). Sensitivity analyses showed that our estimates were unaffected by adjustments for whether the exposures were measured before or during the pandemic. Still, it is possible that the mechanism linking study environment factors to mental and musculoskeletal health could have been different during the pandemic. For instance, physical study environment may have had larger effects on the outcomes, had the students been followed during a time-period when they spent more time on campus. Thus, it is not certain to what extent these results, especially those regarding the physical

For peer review only

study environment, will generalize to non-pandemic periods. Further, our sample was restricted to certain universities, and to participants agreeing to participate, and does not fully represent the overall Swedish student population. It is therefore unclear how well these results may generalize to other populations. We believe, however, that the associations observed in this study are similar to those in other populations at other time-periods, a belief supported by the alignment of our results to those of prior research conducted in different populations before the COVID-19 pandemic (16-20, 23-26).

Conclusions

Several aspects of the study environment were associated with the incidence of mental health problems and activity-limiting musculoskeletal problems in our sample of Swedish university students, after controlling for multiple potential confounders.

For peer review only

Figure 1. Flowchart of the inclusion of participants. MHC = Mental health cohort; MSKHC = Musculoskeletal health cohort.

Acknowledgements

We thank Pierre Côté, Klara Edlund and Tobias Sundberg for their important contributions in planning and performing the SUN-study data collection. We would also like to express our appreciation to all the participating students for their contribution to the SUN-study.

Funding

This research project was funded by the Swedish Research Council for Health, Working Life and Welfare (FORTE), grant number FORTE2018-00402. The SUN-study also received financial support from the Public Health Agency of Sweden.

Competing interests

The authors report no competing interests.

Contributorship statement

Conceptualization – Fred Johansson, Jessica Billquist, Hanna Andreasson, and Eva Skillgate

Data curation – Fred Johansson

Formal Analysis – Fred Johansson

Funding acquisition – Eva Skillgate

Investigation – Fred Johansson, Clara Onell, and Eva Skillgate

Methodology – Fred Johansson and Eva Skillgate

Project administration – Eva Skillgate

For peer review only

Supervision – Anne H Berman and Eva Skillgate

Writing – original draft – Fred Johansson, Jessica Billquist, Hanna Andreasson and Eva Skillgate

Writing – review & editing – Fred Johansson, Jessica Billquist, Hanna Andreasson, Irene Jensen, Clara Onell, Anne H Berman, and Eva Skillgate

Data Availability Statement

The dataset analyzed in the current study is not publicly available due to secondary confidentiality and privacy of the participants.

Ethics Approval

The study was approved by the Swedish Ethical Review Authority (2019-03276), and all participants provided informed consent electronically before entering the study.

References

1. Mokdad AH, Forouzanfar MH, Daoud F, Mokdad AA, El Bcheraoui C, Moradi-Lakeh M, et al. Global burden of diseases, injuries, and risk factors for young people's health during 1990-2013: a systematic analysis for the Global Burden of Disease Study 2013. Lancet. 2016;387(10036):2383-401.
2. Rotenstein LS, Ramos MA, Torre M, Segal JB, Peluso MJ, Guille C, et al. Prevalence of Depression, Depressive Symptoms, and Suicidal Ideation Among Medical Students: A Systematic Review and Meta-Analysis. JAMA: Journal of the American Medical Association. 2016;316(21):2214-36.
3. Ibrahim AK, Kelly SJ, Adams CE, Glazebrook C. A systematic review of studies of depression prevalence in university students. J Psychiatr Res. 2013;47(3):391-400.
4. Sheldon E, Simmonds-Buckley M, Bone C, Mascarenhas T, Chan N, Wincott M, et al. Prevalence and risk factors for mental health problems in university undergraduate students: A systematic review with meta-analysis. J Affect Disord. 2021;287:282-92.
5. Auerbach RP, Alonso J, Axinn WG, Cuijpers P, Ebert DD, Green JG, et al. Mental disorders among college students in the World Health Organization World Mental Health Surveys. Psychol Med. 2016;46(14):2955-70.
6. Keyes CL, Eisenberg D, Perry GS, Dube SR, Kroenke K, Dhingra SS. The relationship of level of positive mental health with current mental disorders in predicting suicidal behavior and academic impairment in college students. J Am Coll Health. 2012;60(2):126-33.
7. Rudman A, Gustavsson JP. Burnout during nursing education predicts lower occupational preparedness and future clinical performance: a longitudinal study. Int J Nurs Stud. 2012;49(8):988-1001.
8. Kim HJ, Boo S, Meeker TJ. Pain Prevalence, Management and Interference Among University Students in South Korea: An Exploratory Cross-Sectional Study. J Pain Res. 2021;14:2423-31.
9. Robertson D, Kumbhare D, Nolet P, Srbely J, Newton G. Associations between low back pain and depression and somatization in a Canadian emerging adult population. J Can Chiropr Assoc. 2017;61(2):96-105.

For peer review only

10. El Ansari W, Stock C, Snelgrove TUSHGS, Hu X, Parke S, Davies S, et al. Feeling Healthy? A Survey of Physical and Psychological Wellbeing of Students from Seven Universities in the UK. Int J Environ Res Public Health. 2011;8(5):1308-23.
11. Serbic D, Friedrich C, Murray R. Psychological, social and academic functioning in university students with chronic pain: A systematic review. J Am Coll Health. 2021:1-15.
12. Bair MJ, Robinson RL, Katon W, Kroenke K. Depression and Pain Comorbidity: A Literature Review. Arch Intern Med. 2003;163(20):2433-45.
13. Kroenke K, Wu J, Bair MJ, Krebs EE, Damush TM, Tu W. Reciprocal relationship between pain and depression: a 12-month longitudinal analysis in primary care. J Pain. 2011;12(9):964-73.
14. Bondesson E, Larrosa Pardo F, Stigmar K, Ringqvist Å, Petersson IF, Jöud A, et al. Comorbidity between pain and mental illness - Evidence of a bidirectional relationship. Eur J Pain. 2018;22(7):1304-11.
15. Gröjer A, Wikström Öbrand I, Elenäs J, Gillström P, Dryler H, Eriksson G. Studentspegeln 2016. 2017.
16. Pascoe EA, Smart Richman L. Perceived discrimination and health: a meta-analytic review. Psychol Bull. 2009;135(4):531-54.
17. Schmitt MT, Branscombe NR, Postmes T, Garcia A. The consequences of perceived discrimination for psychological well-being: a meta-analytic review. Psychol Bull. 2014;140(4):921-48.
18. Brown TT, Partanen J, Chuong L, Villaverde V, Chantal Griffin A, Mendelson A. Discrimination hurts: The effect of discrimination on the development of chronic pain. Soc Sci Med. 2018;204:1-8.
19. Barker ET, Howard AL, Villemaire-Krajden R, Galambos NL. The Rise and Fall of Depressive Symptoms and Academic Stress in Two Samples of University Students. J Youth Adolesc. 2018;47(6):1252-66.
20. Nerdrum P, Rustøen T, Helge Rønnestad M. Psychological Distress Among Nursing, Physiotherapy and Occupational Therapy Students: A Longitudinal and Predictive Study. Scandinavian Journal of Educational Research. 2009;53(4):363-78.
21. Grimby-Ekman A, Andersson EM, Hagberg M. Analyzing musculoskeletal neck pain, measured as present pain and periods of pain, with three different regression models: a cohort study. BMC Musculoskelet Disord. 2009;10:73.
22. Leigh-Hunt N, Bagguley D, Bash K, Turner V, Turnbull S, Valtorta N, et al. An overview of systematic reviews on the public health consequences of social isolation and loneliness. Public Health. 2017;152:157-71.
23. Santini ZI, Pisinger VSC, Nielsen L, Madsen KR, Nelausen MK, Koyanagi A, et al. Social Disconnectedness, Loneliness, and Mental Health Among Adolescents in Danish High Schools: A Nationwide Cross-Sectional Study. Front Behav Neurosci. 2021;15:632906.
24. Hoogendoorn WE, van Poppel MN, Bongers PM, Koes BW, Bouter LM. Systematic review of psychosocial factors at work and private life as risk factors for back pain. Spine (Phila Pa 1976). 2000;25(16):2114-25.
25. Ogunlana MO, Govender P, Oyewole OO. Prevalence and patterns of musculoskeletal pain among undergraduate students of occupational therapy and physiotherapy in a South African university. Hong Kong Physiother J. 2021;41(1):35-43.
26. Gheysvandi E, Dianat I, Heidarimoghadam R, Tapak L, Karimi-Shahanjarini A, Rezapur-Shahkolai F. Neck and shoulder pain among elementary school students: prevalence and its risk factors. BMC Public Health. 2019;19(1):1299.
27. Edlund K, Sundberg T, Johansson F, Onell C, Rudman A, Holm LW, et al. Sustainable UNiversity Life (SUN) study: protocol for a prospective cohort study of modifiable risk and prognostic factors for mental health problems and musculoskeletal pain among university students. BMJ Open. 2022;12(4):e056489.
28. Henry JD, Crawford JR. The short-form version of the Depression Anxiety Stress Scales (DASS-21): construct validity and normative data in a large non-clinical sample. Br J Clin Psychol. 2005;44(Pt 2):227-39.

For peer review only

29. Alfonsson S, Wallin E, Maathz P. Factor structure and validity of the Depression, Anxiety and Stress Scale-21 in Swedish translation. J Psychiatr Ment Health Nurs. 2017;24(2-3):154-62.
30. Kuorinka I, Jonsson B, Kilbom A, Vinterberg H, Biering-Sørensen F, Andersson G, et al. Standardised Nordic questionnaires for the analysis of musculoskeletal symptoms. Appl Ergon. 1987;18(3):233-7.
31. Hefner J, Eisenberg D. Social Support and Mental Health Among College Students. Am J Orthopsychiatry. 2009;79(4):491-9.
32. Bewick B, Koutsopoulou G, Miles J, Slaa E, Barkham M. Changes in undergraduate students' psychological well-being as they progress through university. Studies in Higher Education. 2010;35(6):633-45.
33. Beiter R, Nash R, McCrady M, Rhoades D, Linscomb M, Clarahan M, et al. The prevalence and correlates of depression, anxiety, and stress in a sample of college students. J Affect Disord. 2015;173:90-6.
34. Hernan MA, Robins JM. Causal Inference: What If. 1st ed. Boca Raton: Chapman & Hall/CRC; 2020.
35. Allison PD. Discrete-Time Methods for the Analysis of Event Histories. Sociol Methodol. 1982;13:61-98.
36. Lu H, Cole SR, Howe CJ, Westreich D. Toward a Clearer Definition of Selection Bias When Estimating Causal Effects. Epidemiology. 2022;33(5):699-706.
37. Smith LH, VanderWeele TJ. Bounding Bias Due to Selection. Epidemiology. 2019;30(4):509-16.
38. Johansson F, Côté P, Hogg-Johnson S, Skillgate E. Depression, anxiety and stress among Swedish university students during the second and third waves of COVID-19: A cohort study. Scandinavian Journal of Public Health. 2021:14034948211031402.
39. Johansson F, Cote P, Hogg-Johnson S, Rudman A, Holm LW, Grotle M, et al. Depression, anxiety and stress among Swedish university students before and during six months of the COVID-19 pandemic: A cohort study. Scand J Public Health. 2021;49(7):741-9.
40. Berman AH, Bendtsen M, Molander O, Lindfors P, Lindner P, Granlund L, et al. Compliance with recommendations limiting COVID-19 contagion among university students in Sweden: associations with self-reported symptoms, mental health and academic self-efficacy. Scand J Public Health. 2022;50(1):70-84.

For peer review only

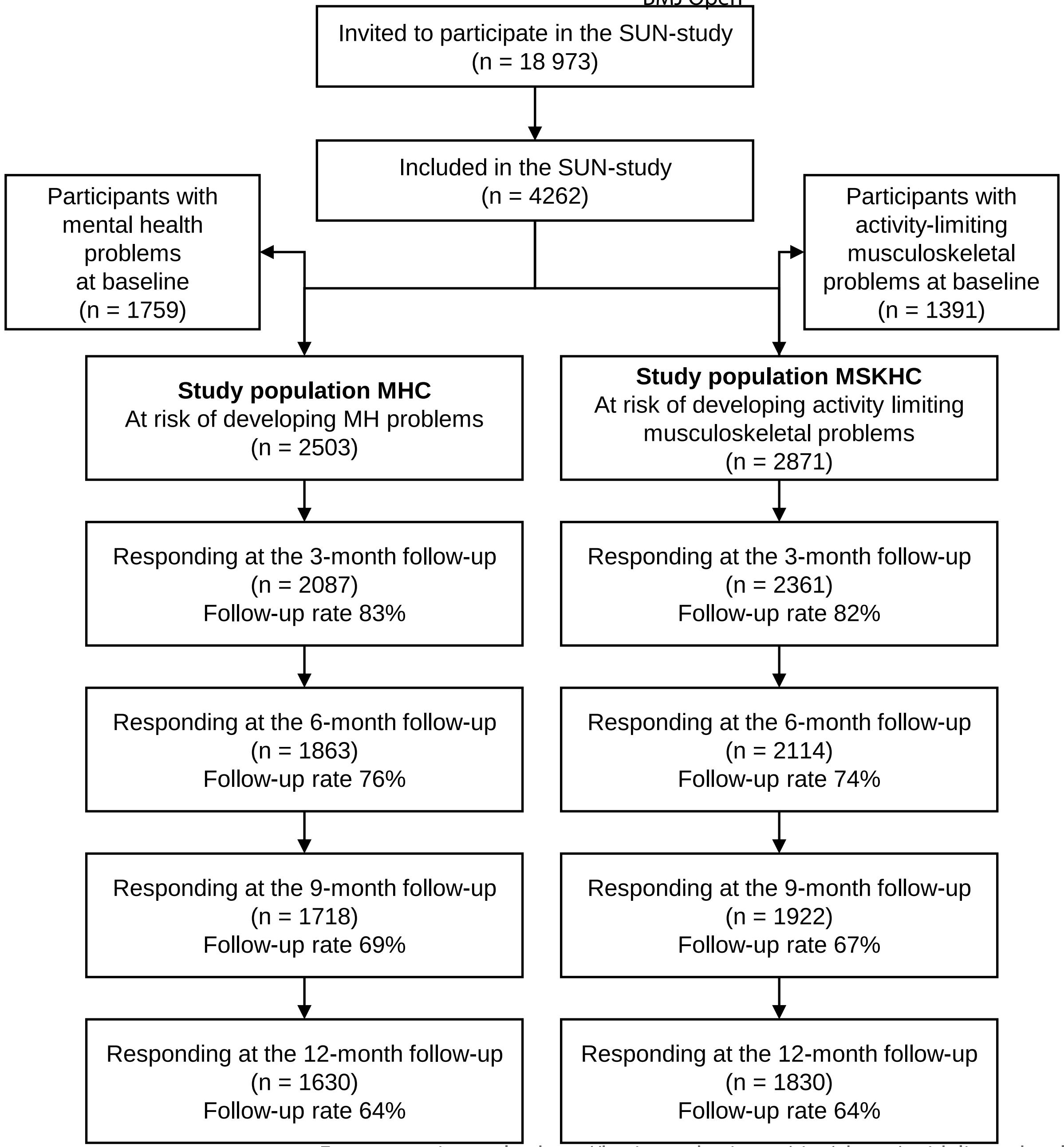
Invited to participate in the SUN-study
(n = 18 973)
Included in the SUN-study
(n = 4262)
Participants with mental health problems at baseline
(n = 1759)
Participants with activity-limiting musculoskeletal problems at baseline
(n = 1391)
Study population MHC
At risk of developing MH problems
(n = 2503)
Study population MSKHC
At risk of developing activity limiting musculoskeletal problems
(n = 2871)
Responding at the 3-month follow-up
(n = 2087)
Follow-up rate 83%
Responding at the 3-month follow-up
(n = 2361)
Follow-up rate 82%
Responding at the 6-month follow-up
(n = 1863)
Follow-up rate 76%
Responding at the 6-month follow-up
(n = 2114)
Follow-up rate 74%
Responding at the 9-month follow-up
(n = 1718)
Follow-up rate 69%
Responding at the 9-month follow-up
(n = 1922)
Follow-up rate 67%
Responding at the 12-month follow-up
(n = 1630)
Follow-up rate 64%
Responding at the 12-month follow-up
(n = 1830)
Follow-up rate 64%

Supplemental material

Johansson, F., Billquist, J., Andreasson, H., Jensen, I., Onell, C., Berman, A. H., Skillgate, E. Study Environment and the Incidence of Mental Health Problems and Activity-Limiting Musculoskeletal Problems among University Students: The SUN Cohort Study

eTable 1. Number of events and participants at risk at each time-period.

eTable 2. COVID-19 adjusted hazard ratios of mental health problems and activity-limiting musculoskeletal problems in any body location comparing exposed to unexposed

eTable 3. Gender specific hazard ratios of mental health problems and activity-limiting musculoskeletal problems in any body location comparing exposed to unexposed

eTable 4. Association between baseline exposure and dropout at the 12-month follow-up

eTable 5. E-values for selection bias giving the minimum association between the outcome and missingness among the unexposed needed to move the HR estimates to the null

eTable 6. Dose response models for high study pace and low social cohesion with each response category as separate exposure level

For peer review only

eTable 1. Number of events and participants at risk at each time-period

	Mental health cohort			Musculoskeletal health cohort		
Time-period	*Events (n)*	*At risk (n)* [a]	*Hazard* [b]	*Events (n)*	*At risk (n)* [a]	*Hazard* [b]
0-3 months	502	2087	0.24	475	2361	0.20
3-6 months	180	1436	0.13	234	1711	0.13
6-9 months	133	1163	0.11	142	1357	0.10
9-12 months	94	997	0.09	121	1184	0.10

[a] Number are not equal to the analytic sample since not all participants were observed at all time-points.

[b] The hazard equals the probability of the event conditional on not having had the event earlier (Events / At risk).

For peer review only

**eTable 2.** COVID-19 adjusted [a] hazard ratios of mental health problems and activity-limiting musculoskeletal problems in any body location comparing exposed to unexposed		
	**Mental health problems**	**Activity-limiting musculoskeletal problems**
	Adjusted HR [b] *(95% CI)*	*Adjusted HR [b]* *(95% CI)*
Discrimination	1.76 (1.41 – 2.21)	1.38 (1.12 – 1.72)
High study pace	1.70 (1.48 – 1.95)	1.25 (1.09 – 1.43)
Low social cohesion	1.51 (1.29 – 1.76)	1.08 (0.93 – 1.26)
Poor physical environment	1.20 (0.99 – 1.46)	1.20 (1.01 – 1.43)

[a] these analyses include a dummy coded covariate indicating whether the baseline survey, where the exposures were measured, were collected before or after March 13, 2020, that is considered the start of the COVID-19 pandemic in Sweden.

[b] Adjusted for gender, age, living situation, education type, year of study, place of birth and highest level of parental education and covid-19 pandemic at baseline.

For peer review only

eTable 3. Gender specific hazard ratios of mental health problems and activity-limiting musculoskeletal problems in any body location comparing exposed to unexposed

	**Mental health problems** *Adjusted HR [a]* *(95% CI)*		**Activity-limiting musculoskeletal problems** *Adjusted HR [a]* *(95% CI)*	
	Women (n = 1263 [b])	Men (n = 927 [c])	Women (n = 1491 [d])	Men (n = 1007 [e])
Discrimination	1.79 (1.39 – 2.31)	1.85 (1.16 – 2.95)	1.31 (1.01 - 1.69)	1.58 (1.08 – 2.33)
High study pace	1.50 (1.27 – 1.78)	2.11 (1.67 – 2.66)	1.29 (1.09 - 1.53)	1.15 (0.92 – 1.43)
Low social cohesion	1.40 (1.15 – 1.71)	1.81 (1.41 – 2.32)	1.14 (0.94 – 1.38)	1.01 (0.79 – 1.29)
Poor physical environment	1.12 (0.89 – 1.42)	1.31 (0.94 – 1.83)	1.18 (0.95 – 1.46)	1.28 (0.95 – 1.71)

[a] Adjusted for age, living situation, education type, year of study, place of birth and highest level of parental education.

[b]1427 women were included in the cohort at baseline, but 164 did not provide any follow-up assessments, giving an analytic sample size of 1263.

[c] 1065 men were included in the cohort at baseline, but 138 did not provide any follow-up assessments, giving an analytic sample size of 927.

[d] 1698 women were included in the cohort at baseline, but 206 did not provide any follow-up assessments, giving an analytic sample size of 1491

[e] 1057 men were included in the cohort at baseline, but 150 did not provide any follow-up assessments, giving an analytic sample size of 1007.

For peer review only

**eTable 4**. Association between baseline exposure and dropout at the 12-month follow-up		
	**Mental health cohort**	**Musculoskeletal health cohort**
	RR (95% CI)	*RR (95% CI)*
Discrimination	1.15 (0.95 - 1.39)	1.27 (1.10 – 1.48)
High study pace	0.98 (0.88 – 1.09)	1.03 (0.94 – 1.14)
Low social cohesion	1.05 (0.92 – 1.20)	1.07 (0.96 – 1.19)
Poor physical environment	1.10 (0.95 – 1.28)	1.18 (1.04 – 1.33)
RR: Risk ratio		

**eTable 5**. E-values for selection bias giving the minimum association between the outcome and missingness among the unexposed needed to move the HR estimates [a] to the null [b]		
	**Mental health cohort**	**Musculoskeletal health cohort**
	RR (95% CI)	*RR (95% CI)*
Discrimination	1.47	1.26
High study pace	1.44	1.16
Low social cohesion	1.33	1.05
Poor physical environment	1.13	1.13
[a] This refers to the adjusted estimates presented in Table 5<br>[b] Assuming that missingness is higher for persons with the outcome among both exposed and unexposed<br>RR: Risk ratio		

For peer review only

**eTable 6**. Dose response models for high study pace and low social cohesion with each response category as a separate exposure level				
	**Mental health cohort**		**Musculoskeletal health cohort**	
	No. exp [a]	*Adjusted HR (95% CI)* [b]	*No. exp* [a]	*Adjusted HR (95% CI)* [b]
**High study pace**				
I feel that I have energy and time to cope with the study pace	1079	Ref.	1044	Ref.
The study pace is high. I do my best, but I am afraid that it is not enough	967	1.64 (1.43 - 1.88)	1201	1.20 (1.05 - 1.37)
The study pace breaks me. We get more assignments than I have time for, and I feel that I cannot make it	41	3.28 (2.25 - 4.77)	116	1.50 (1.13 - 1.98)
**Low social cohesion**				
The cohesion is pretty good. We help each other with the studies and there is always someone in the class to talk to	1662	Ref.	1779	Ref.
I do not think that our cohesion is good. We work independently, and it feels like it is not worth the inconvenience to try to change it	366	1.49 (1.27 - 1.70)	506	1.07 (0.92 - 1.25)
We do not have any cohesion, and no one takes any responsibility over our groupwork or the fellowship	26	2.12 (1.30 - 3.45)	45	1.14 (0.72 - 1.80)

[a] Refers to the number of exposed participants in the analytic sample at baseline.
[b] Adjusted for age, living situation, education type, year of study, place of birth and highest level of parental education.

For peer review only

STROBE Statement—Checklist of items that should be included in reports of ***cohort studies***

	Item No	Recommendation	Page No
**Title and abstract**	1	(*a*) Indicate the study's design with a commonly used term in the title or the abstract<br>(*b*) Provide in the abstract an informative and balanced summary of what was done and what was found	1-2
**Introduction**			
Background/rationale	2	Explain the scientific background and rationale for the investigation being reported	4-5
Objectives	3	State specific objectives, including any prespecified hypotheses	5
**Methods**			
Study design	4	Present key elements of study design early in the paper	6
Setting	5	Describe the setting, locations, and relevant dates, including periods of recruitment, exposure, follow-up, and data collection	6
Participants	6	(*a*) Give the eligibility criteria, and the sources and methods of selection of participants. Describe methods of follow-up<br>(*b*) For matched studies, give matching criteria and number of exposed and unexposed	6
Variables	7	Clearly define all outcomes, exposures, predictors, potential confounders, and effect modifiers. Give diagnostic criteria, if applicable	6-8
Data sources/ measurement	8*	For each variable of interest, give sources of data and details of methods of assessment (measurement). Describe comparability of assessment methods if there is more than one group	6-8
Bias	9	Describe any efforts to address potential sources of bias	9
Study size	10	Explain how the study size was arrived at	-
Quantitative variables	11	Explain how quantitative variables were handled in the analyses. If applicable, describe which groupings were chosen and why	9
Statistical methods	12	(*a*) Describe all statistical methods, including those used to control for confounding<br>(*b*) Describe any methods used to examine subgroups and interactions<br>(*c*) Explain how missing data were addressed<br>(*d*) If applicable, explain how loss to follow-up was addressed<br>(*e*) Describe any sensitivity analyses	9
**Results**			
Participants	13*	(a) Report numbers of individuals at each stage of study—eg numbers potentially eligible, examined for eligibility, confirmed eligible, included in the study, completing follow-up, and analysed<br>(b) Give reasons for non-participation at each stage<br>(c) Consider use of a flow diagram	10 and figure 1
Descriptive data	14*	(a) Give characteristics of study participants (eg demographic, clinical, social) and information on exposures and potential confounders<br>(b) Indicate number of participants with missing data for each variable of interest<br>(c) Summarise follow-up time (eg, average and total amount)	10, table 1-4
Outcome data	15*	Report numbers of outcome events or summary measures over time	Table4

For peer review only - http://bmjopen.bmj.com/site/about/guidelines.xhtml

Main results	16	(*a*) Give unadjusted estimates and, if applicable, confounder-adjusted estimates and their precision (eg, 95% confidence interval). Make clear which confounders were adjusted for and why they were included<br>(*b*) Report category boundaries when continuous variables were categorized<br>(*c*) If relevant, consider translating estimates of relative risk into absolute risk for a meaningful time period	Table 4 and 5
Other analyses	17	Report other analyses done—eg analyses of subgroups and interactions, and sensitivity analyses	11
**Discussion**			
Key results	18	Summarise key results with reference to study objectives	11
Limitations	19	Discuss limitations of the study, taking into account sources of potential bias or imprecision. Discuss both direction and magnitude of any potential bias	12-13
Interpretation	20	Give a cautious overall interpretation of results considering objectives, limitations, multiplicity of analyses, results from similar studies, and other relevant evidence	14
Generalisability	21	Discuss the generalisability (external validity) of the study results	14
**Other information**			
Funding	22	Give the source of funding and the role of the funders for the present study and, if applicable, for the original study on which the present article is based	19

*Give information separately for exposed and unexposed groups.

Note: An Explanation and Elaboration article discusses each checklist item and gives methodological background and published examples of transparent reporting. The STROBE checklist is best used in conjunction with this article (freely available on the Web sites of PLoS Medicine at http://www.plosmedicine.org/, Annals of Internal Medicine at http://www.annals.org/, and Epidemiology at http://www.epidem.com/). Information on the STROBE Initiative is available at http://www.strobe-statement.org.

For peer review only

For peer review only - http://bmjopen.bmj.com/site/about/guidelines.xhtml
